# Study on the Binding Interaction of the α,α′,δ,δ′-Tetramethylcucurbit[6]uril With Biogenic Amines in Solution and the Solid State

**DOI:** 10.3389/fchem.2018.00289

**Published:** 2018-07-17

**Authors:** Liguo Yang, Jinglan Kan, Xin Wang, Yonghui Zhang, Zhu Tao, Qingyun Liu, Fang Wang, Xin Xiao

**Affiliations:** ^1^College of Chemistry and Environmental Engineering, Anyang Institute of Technology, Anyang, China; ^2^Key Laboratory of Molecular and Nano Probes, Ministry of Education, Collaborative Innovation Center of Functionalized Probes for Chemical Imaging in Universities of Shandong, College of Chemistry, Chemical Engineering and Materials Science, Shandong Normal University, Jinan, China; ^3^Key Laboratory of Macrocyclic and Supramolecular Chemistry of Guizhou Province, Guizhou University, Guiyang, China; ^4^College of Chemistry and Environmental Engineering, Shandong University of Science and Technology, Qingdao, China

**Keywords:** biogenic amines, cucurbit[6]uril, crystal structure, NMR spectra, host-guest interactions

## Abstract

^1^H NMR spectroscopy and MALDI-TOF mass spectrometry were utilized to examine the binding interaction of α,α′,δ,δ′-tetramethylcucurbit[6]uril (TMeQ[6]) and six biogenic amines (spermine, spermidine, 2-phenethylamine, tyramine, histamine, and tryptamine). Their ^1^H NMR spectra both at pD = 7 and pD = 3 revealed that four biogenic amines (spermine, spermidine, 2-phenethylamine, and histamine) can fit in the TMeQ[6] cavity, respectively, and other biogenic amines were located outside of the TMeQ[6] portal. In addition, a solid-state evaluation with single-crystal X-ray diffraction analysis showed the binding interaction of spermine, spermidine, 2-phenethylamine, and tyramine with TMeQ[6].

## Introduction

Biogenic amines (BAs) belong to a group of low-molecular weight nitrogen-containing organic compounds with remarkable biological activity. BAs are categorized by structure into three groups: aliphatic amines (e.g., spermine and spermidine), aromatic amines (e.g., tyramine and phenylethylamine), and heterocyclic amines (e.g., histamine and tryptamine) (Sentellas et al., 2016). These amines can develop via the thermal or enzymatic decarboxylation of amino acids due to the storage and handling of food, and increased levels of BAs suggest spoilage of food. Thus, BAs are pivotal markers for food poisoning (Santos, 1996; Shalaby, 1996). Since biogenic amines also take part in critical physiological functions, the determination of the BA content in food is important.

Numerous methods, such as chromatography (LC, HPLC, GC, and ion exchange), electrophoretic techniques, fluorescence, and UV–Vis spectroscopy (Khuhawar and Qureshi, 2001; Reinemann et al., 2009; Zotou and Notou, 2012; Mohammed et al., 2016; Sun et al., 2017), have been reported for BA detection. Furthermore, based on selective recognition through non-covalent interactions between a receptor unit and an analyte, the supramolecular-complex method has been used to identify BAs. Following this strategy, various macrocycles (cyclodextrins, calix[4] arenes, and crown ethers) have been used as hosts, and detection methods are often based on changes in the host (Galian et al., 1998; Saaid et al., 2010; Jiang et al., 2011; Galego et al., 2016; Remy et al., 2017; Zhou et al., 2017). Thus, studies on the interactions between these macrocycles and BAs have been reported in the past few years (Ballistreri et al., 2003; Zhou et al., 2014; Gattuso et al., 2015; D'Urso et al., 2016).

Cucurbit[*n*]urils (Q[*n*]), which are pumpkin-shaped macrocyclic host molecules, have garnered substantial awareness since they can tightly bind to different biological molecules in the solid state and aqueous solution (Kim et al., 2000; Day et al., 2002; Li et al., 2016; Chen et al., 2017a; Gao et al., 2017a,b; Wang et al., 2017). Several examples of supramolecular assemblies of Q[*n*] and certain BAs have been reported (Masson et al., 2009; Chen et al., 2017b). Kim et al. utilized atomic force microscopy and identified the interaction between Q[6] and spermine, and they additionally utilized isothermal calorimetry, nuclear magnetic resonance (NMR), and single-crystal X-ray diffraction to examine the complexation of cyclohexanocucurbit[6]uril with spermine (Kim et al., 2009). Danylyuk et al. reported the solid-state supramolecular assemblies of Q[6] and tryptamine (Danylyuk and Fedin, 2012). Notably, reports on supramolecular assemblies of Q[*n*] and BAs are scarce.

**Figure 1:**
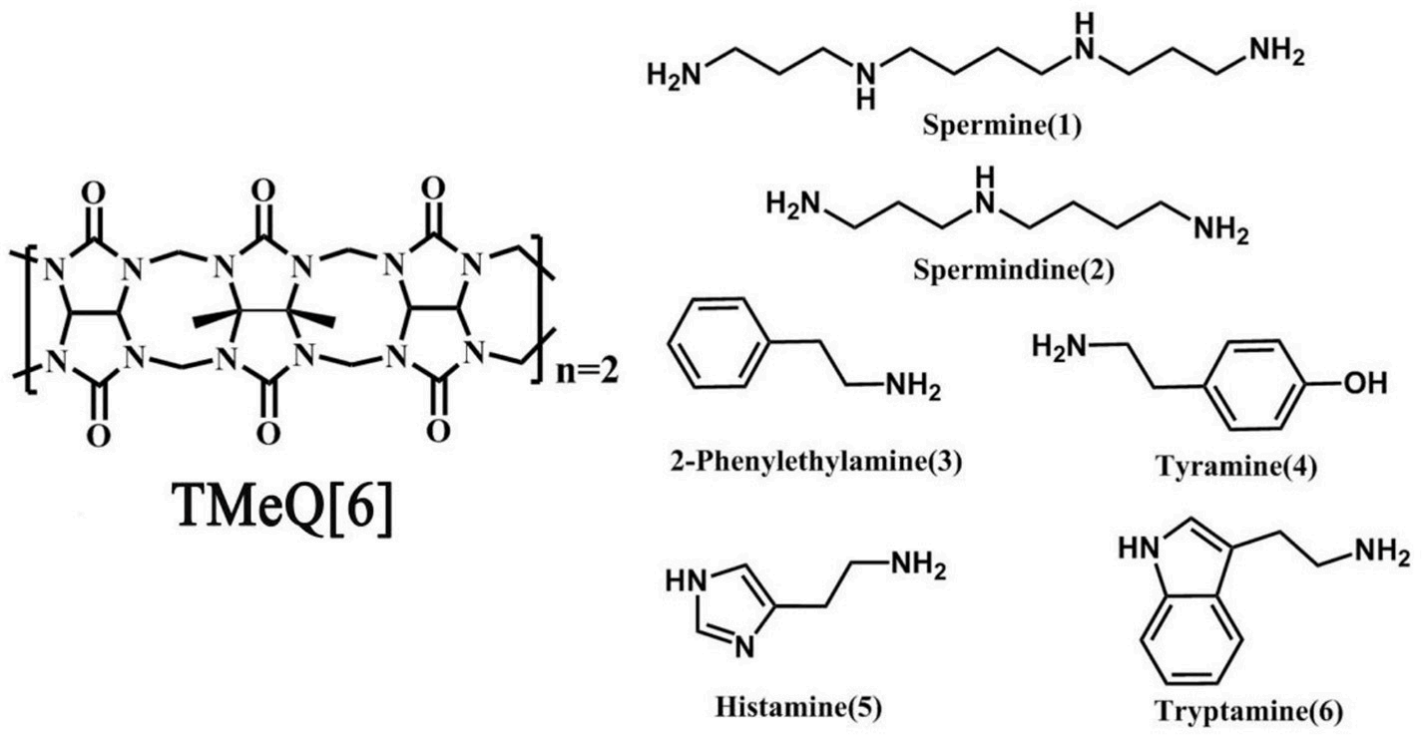
Chemical structure of Host **(Left)** and guest molecules **(Right)** utilized in this evaluation.

Q[6] with four methyl groups on the α, α′, δ, and δ′ positions (tetramethyl-cucurbit[6]uril, TMeQ[6]) was synthesized (Figure 1). The introduction of four methyl groups can increase molecular polarity while decreasing molecular symmetry, allowing easy reaction with other agents in aqueous media. Thus, a set of new host–guest complexes of TMeQ[6] has been synthesized (Xiao et al., 2013, 2015; Yang et al., 2014, 2016; Gao et al., 2016; Shan et al., 2017). To study the binding conduct of BAs and TMeQ[6], we methodically utilized ^1^H NMR spectroscopy, matrix-assisted laser desorption/ionization–time-of-flight (MALDI-TOF) mass spectrometry, and X-ray crystallography to examine the interaction of six BA guests; namely, spermine (**1**), spermidine (**2**), 2-phenethylamine (**3**), tyramine (**4**), histamine (**5**), and tryptamine (**6**), with TMeQ[6].

## Results and discussions

### Nature of binding in aqueous solution

The interactions between the host TMeQ[6] and the biogenic amine guests in aqueous solution were examined by NMR spectroscopy. Then, ^1^H NMR spectroscopy was conducted on each biogenic amine guest with TMeQ[6], and the NMR assignments were made through ^1^H-^1^H COSY (Figures S1–S6). The ambiguously binding behavior will be discussed.

**Figure 2:**
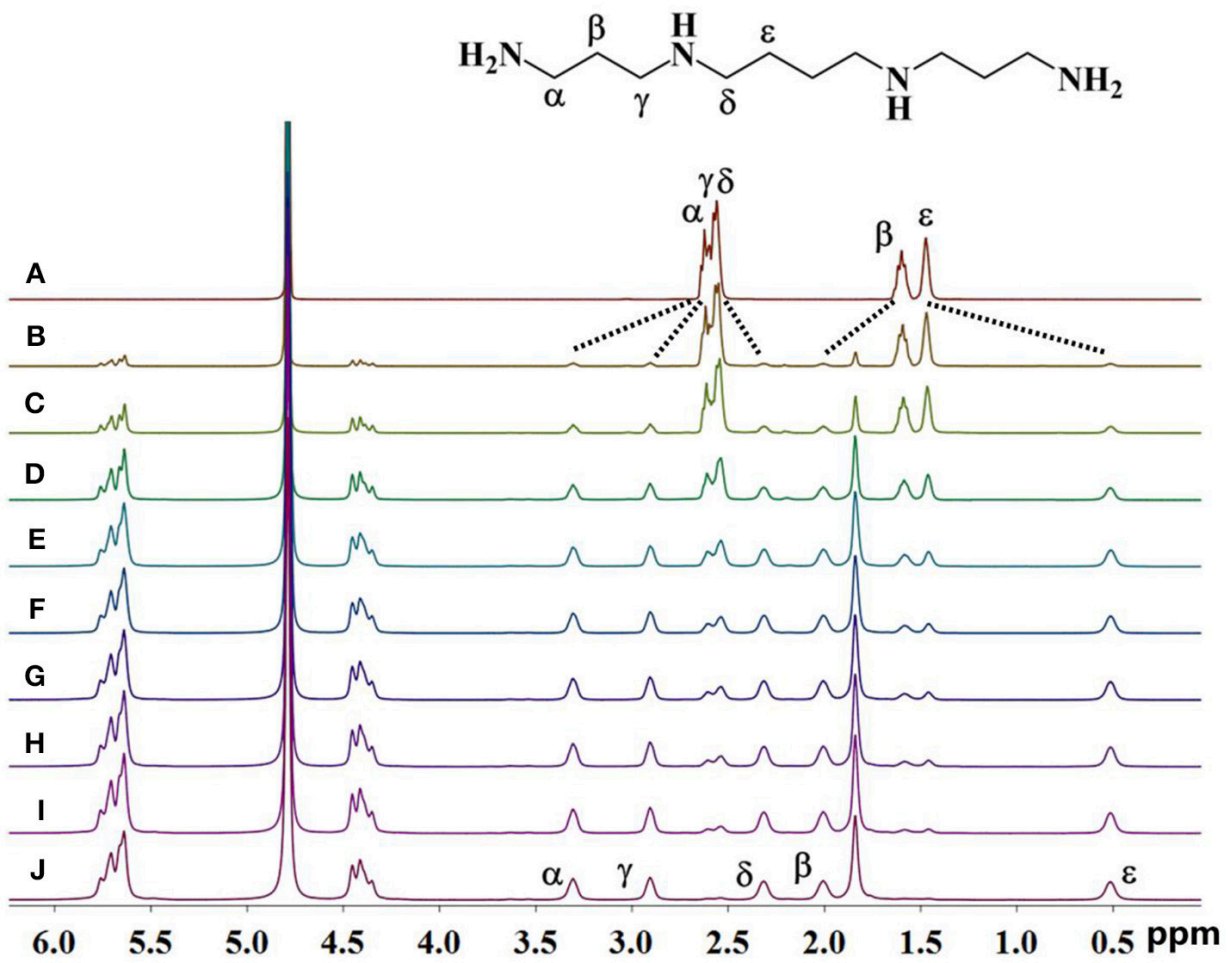
^1^H NMR spectra (400 MHz, D_2_O) of guest **1** without **(A)** and with **(B)** 0.10, **(C)** 0.18, **(D)** 0.25, **(E)** 0.39, **(F)** 0.58, **(G)** 0.67, **(H)** 0.78, **(I)** 0.98, and **(J)** 1.05 equiv. of TMeQ[6] **(B–J)**.

As shown in Figure 2, upon continuous addition of additional hosts, the signals of free guest **1** gradually disappeared, and new signals of the supramolecular complexes appeared as a result of the binding behavior. The resonances of α, γ, and β of guest **1** moved downfield by 0.70, 0.37, and 0.60 ppm, respectively, whereas the resonances of δ and ε of guest **1** shifted upfield by 0.40 and 0.96 ppm, respectively. This observation indicates that only a portion of guest **1** is buried deeply inside the host, forming a stable inclusion complex TMeQ[6]@ **1**. The α, γ, and β protons are located outside of TMeQ[6], whereas the δ and ε protons of guest **1** are located in TMeQ[6].

**Figure 3:**
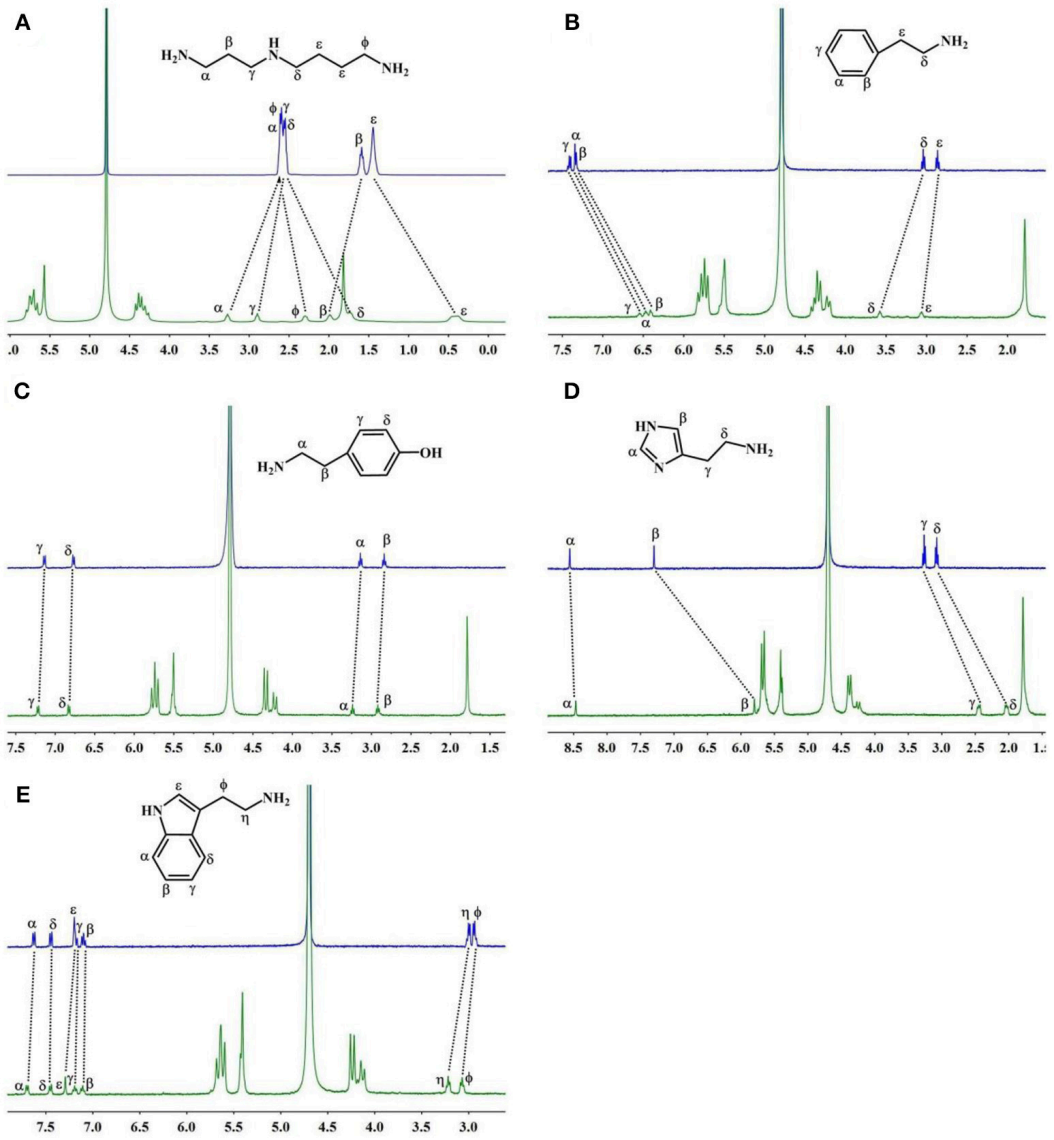
^1^H NMR spectra (400 MHz, D_2_O) of **(A–E)** guests **2**–**6** without (up) and with 1.1 equiv of TMeQ[6] (down).

The ^1^H NMR spectra in Figure 3A reveal the presence of **2** in neutral D_2_O solution in the absence and presence of 1.1 equiv. of TMeQ[6]. The α, γ, and β proton resonances of guest **2** moved downfield by 0.67, 0.35, and 0.40 ppm, respectively, while the resonances for φ, δ, and ε protons of guest **2** moved upfield by 0.30, 0.81, and 1.04 ppm, respectively. The outcomes suggest that nearly identical to guest **1**, guest **2** is somewhat concealed within the TMeQ[6] cavity and establishes the stable inclusion complex TMeQ[6]@ **2**.

With 1.1 equiv of TMeQ[6] (Figure 3B) the α, β, and γ proton resonances of guest **3** shifted upfield by 0.86, 0.92, and 0.86 ppm, respectively, while those of the ε and δ protons of guest **3** shifted downfield by 0.21 and 0.54 ppm, respectively. The NMR results indicated that the phenyl group of guest **3** is buried in TMeQ[6], whereas the alkyl chain of guest **3** is located outside the TMeQ[6], forming an inclusion complex TMeQ[6]@ **3**.

The ^1^H NMR spectra of guest **4** with 1.1 equiv. of TMeQ[6] are revealed in Figure 3C, and the resonance for the α, β, γ, and δ protons of guest **4** shifted downfield by 0.07, 0.06, 0.06, and 0.04 ppm, respectively. The results show that guest **4** is located outside of TMeQ[6]. Given the presence of the hydroxyl group, guest **4** is not encapsulated in TMeQ[6].

The binding conduct of guest **5** with TMeQ[6] is altered from that of the other guests in this evaluation and is revealed in Figure 3D. The resonances for the α, β, δ, and γ protons of guest **5** shifted upfield by 0.09, 1.50, 1.05, and 0.81 ppm, respectively. This discovery suggested that guest **5** was fully concealed in the cavity of TMeQ[6], verifying the creation of the inclusion complex.

When guest **6** was combined with TMeQ[6], the resonance for its φ and η protons moved downfield by 0.22 and 0.13 ppm, respectively, and the resonance for the protons of the indolyl group did not notably move (Figure 3E). Based on this outcome, due to the large size of guest **6**, it is found outside the portal of TMeQ[6]. All of ^1^H NMR spectroscopy indicate the guest **1** and **2** formed host-guest inclusion complexes that display slow kinetics of exchange on the ^1^H NMR time scale, while guests **3** and **5** show fast kinetics of exchange, and the ^1^H NMR complexation-induced shifts (CIS) of the protons of guests **1**–**6** are listed in Table 1.

**Table 1:** ^1^H NMR complexation-induced shifts (CIS, Δδ/ppm) of **guest 1–6** upon addition of TMeQ[6] in D_2_O at 298 K.

**1**	***Δδ*/ppm**	**2**	***Δδ*/ppm**	**3**	***Δδ*/ppm**
**Protons**		**Protons**		**Protons**	
α	−0.70	α	−0.67	α	0.86
β	−0.60	β	−0.40	β	0.92
γ	−0.37	γ	−0.35	γ	0.86
δ	0.40	δ	0.81	δ	−0.21
ε	0.96	ε	0.30	ε	−0.54
		η	1.04		
**4**	Δδ**/ppm**	**5**	Δδ**/ppm**	**6**	Δδ**/ppm**
**Protons**		**Protons**		**Protons**	
α	−0.07	α	−0.09	α	0.00
β	−0.06	β	−1.50	β	0.00
γ	−0.06	γ	−1.05	γ	0.00
δ	−0.04	δ	0.81	δ	0.00
				ε	0.00
				η	0.13
				φ	0.22

Additional evidence for the creation of the inclusion complexes of TMeQ[6] with guests **1–6** was provided by MALDI-TOF mass spectrometry. In Figure S7, the MALDI-TOF mass spectra show major signals at *m/z* = 1254.28, 1197.97, 1173.08, 1189.18, 1164.00, and 1212.95, which correspond with their calculated data (1254.34, 1197.20, 1173.18, 1189.18, 1163.08, and 1212.21, respectively).

The host–guest interactions of TMeQ[6] with charged BAs were also investigated at pD = 3 by ^1^H NMR spectroscopy (Figures S8–S13). An NMR titration experiment was conducted at pD = 3 and revealed that guests **1**, **2**, **3**, and **5** created an inclusion complex with the TMeQ[6] host, while guests **4** and **6** were found outside of the portal of the TMeQ[6]. This reaction phenomenon is in accord with the titration experiments conducted in D_2_O. The results indicate that BAs can form complexes with TMeQ[6] both in aqueous and acidic solutions.

The interaction of TMeQ[6] with guest **6** was also evaluated by UV absorbance spectrophotometry and fluorescence spectroscopy. Based on the UV absorption spectroscopy outcomes (Figure S14), the addition of TMeQ[6] to guest **6** in buffered solution (pH 7) was joined by a minor rise in the intensity at 218 nm, suggesting a weak interaction of TMeQ[6] and guest **6**. As revealed in Figure S15, guest **6** showed an 358-nm emission peak at an excitation wavelength of 218 nm. The addition of TMeQ[6] with 1:1 stoichiometry induced a minor reduction and bathochromic shift from 358 to 360 nm in fluorescence intensity at 358 nm. These alterations in the emission profile additionally verify the weak host–guest interaction of TMeQ[6] and guest **6** (Mallick et al., 2016). The results of the spectral analysis are consistent with those from the NMR experiments.

### Isothermal titration calorimetry

To improve our comprehension of host–guest interactions of TMeQ[6] and BAs, we performed isothermal titration calorimetry (ITC) experiments at 25°C in 10 mM sodium phosphate (pH 7.0). Table 2 and Figures S16–S20 reveal the equilibrium association constants (*K*_*a*_) and thermodynamic parameters, respectively, for TMeQ[6] and for the BA guests, with the exception of guest **3**. The experimental outcomes showed *K*_a_ values ranging from 10^3^ to 10^7^ M^−1^. Among which TMeQ[6] binds with spermine with the highest binding affinity, which similar to parent cucurbit[6]uril or cucurbit[6]uril derivatives (Mock and Shih, 1986; Kim et al., 2009; Lucas et al., 2011; Wen et al., 2017). In addition, the *K*_a_ constants of guest **1** and **2** with TMeQ[6] are lower than the corresponding *K*_a_ of guest **1** and **2** with cyclohexyl substituted cucurbit[6]uril(CyH_6_Q[6]), suggesting that the substitution impacts the shape of the cavity (Kim et al., 2009; Wen et al., 2017) (Table S1). From the Δ*H*° and *T*Δ*S*° values revealed in Table 2, the intermolecular complexation interactions of the TMeQ[6] host and all guests seem to be compelled by negative enthalpy alterations, while the interactions of TMeQ[6] host and guests **2** and **4** were compelled by negative enthalpy alterations, along with small negative (unfavorable) entropy alterations. The ITC experiment for guest **3** was not successful because of the weak interaction of TMeQ[6] and guest **3**.

**Table 2:** Complex stability constant (*K*_a_), enthalpy (Δ*H*°), entropy changes (*T*Δ*S*°), and Gibbs free energy (Δ*G*°) for TMeQ[6]–guest interactions in buffered solution at pH 7.

**Guest**	***K*_a_ (M^−1^)**	**Δ*H*^°^ (kJ/mol)**	***T*Δ*S*^°^ (kJ/mol)**	**Δ*G*^°^ (kJ/mol)**
1	4.55 × 10^7^	-48.1	1.31	-49.41
2	9.97 × 10^6^	-42.25	-2.306	-39.94
3	5.5 × 10^3^	-11.9	-4.50	-16.4
5	1.59 × 10^5^	-30.71	3.98	-34.69
6	1.23 × 10^4^	-20.5	4.10	-29.60

The binding conduct of TMeQ[6] with BAs in aqueous solution is detailed above. Guests **1** and **2** are favorably encapsulated into TMeQ[6]. Given their long chain, parts of guests **1** and **2** are located outside TMeQ[6]. The phenyl group of guest **3** is completely encapsulated into TMeQ[6], and the alkyl chain of guest **3** cannot be accommodated due to a lack of available space. Guest **4** was not favorably encapsulated into TMeQ[6] due to the presence of the hydrogen bonding of the hydroxyl group of guest **4** and the portal oxygen atoms of TMeQ[6]. For guest **5**, due to the smaller size of the imidazole ring in contrast to that of the phenyl ring of guest **3**, enough space can accommodate the alkyl group of guest **5**; therefore, guests **3** and **5** are favorably encapsulated into TMeQ[6]. By contrast, guest **6** was not encapsulated into TMeQ[6] because of the large size of the benzimidazole group.

### Determination of solid state structures

Notwithstanding a substantial exertion, diffraction-quality crystals to examine the interaction of TMeQ[6] with these six guests in neutral aqueous solutions could not be procured. While protonated biogenic amine molecules are thought to conduct themselves as usual poly-charged cations in HCl aqueous solution, [ZnCl_4_]^2−^ or [CdCl_4_]^2−^ anions are known to act as efficient structure-directing agents in the creation of Q[*n*]-metal coordination polymers (Ni et al., 2014). Therefore, the addition of [CdCl_4_]^2−^ into the TMeQ[6]-biogenic amine system led to acceptable crystals. We obtained single crystals of the complexes TMeQ[6]@**1**, TMeQ[6]@**2**, TMeQ[6]@**3**, and TMeQ[6]@**4** by slow vapor evaporation. However, the attempt to obtain single crystals of TMeQ[6]@**5** and TMeQ[6]@**6** was unsuccessful. Therefore, solid-state single-crystal X-ray crystallography was utilized to examine the binding conduct of TMeQ[6] with guests **1**, **2**, **3**, and **4**. The selected hydrogen bonding (Å) of the complexes is listed in Table S2.

**Figure 4:**
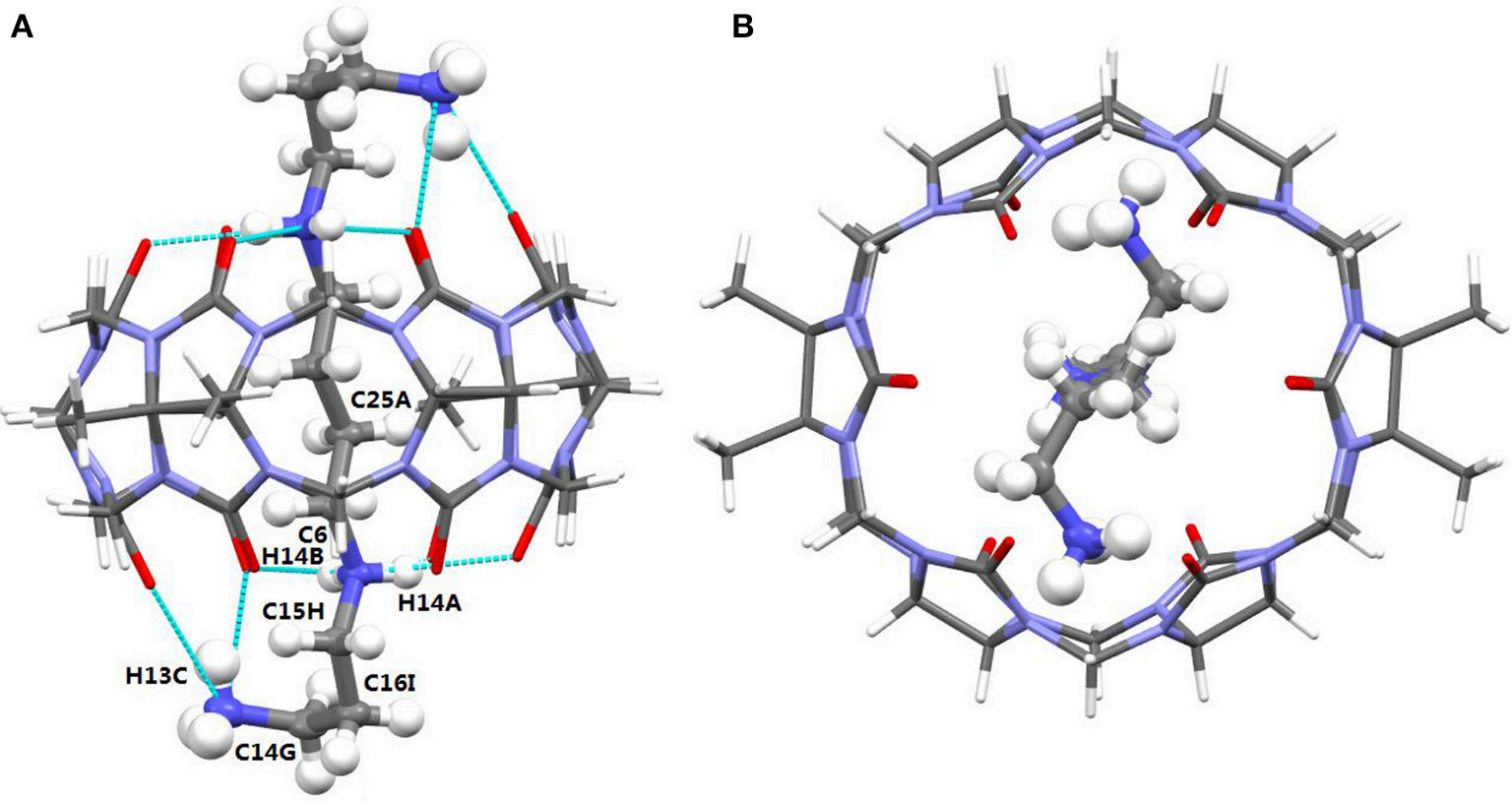
**(A)** Side view and **(B)** top view of the crystal structure of TMeQ[6]@**1**. Solvent water molecules and tetrachloride cadmiumate anions have been excluded for lucidity. O, red; C, gray; N, light blue.

The TMeQ[6]@**1** complex crystallized in the monoclinic systems in space group C 2/c. As shown in Figure 4, guest **1** is encapsulated in TMeQ[6], exiting the back-folded geometry. Moreover, the atoms (C14G, C16I, and C15H) of guest **1** were found in the portal of TMeQ[6], while the atoms C6 and C25A were concealed within the TMeQ[6] cavity, and these structural observations are in accord with the evaluation of the ^1^H NMR spectroscopic data. Guest **1** shows back-folded geometry due to the tight hydrogen bonds between the guest **1** nitrogen atoms and the oxygen atoms of TMeQ[6], which is in accord with the literature (Shan et al., 2017).

**Figure 5:**
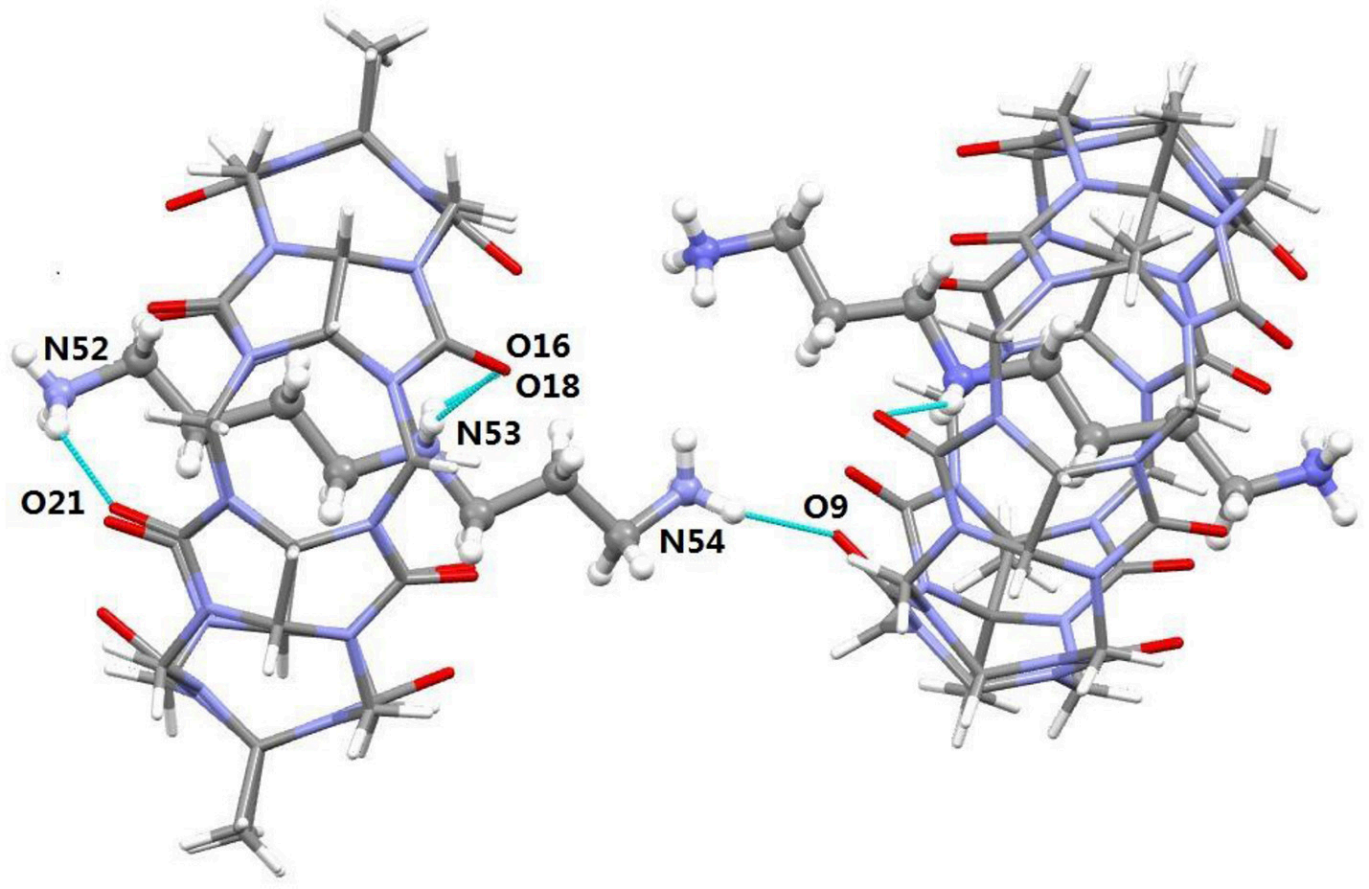
Crystal structure of TMeQ[6]@**2**. Solvent water molecules and tetrachloride zincate anions have been excluded for lucidity. O, red; C, gray; N, light blue.

It was noted that the TMeQ[6]@**2** complex crystallized in the triclinic systems with space group P-1. As revealed in Figure 5, the TMeQ[6]@**2** complex is made up of the TMeQ[6] host, guest **2**, tetrachloride zincate anions, and some water molecules. The asymmetry of the TMeQ[6]@**2** complex results from the asymmetrical structure of guest **2**. Guest **2** is encapsulated in the TMeQ[6] host, the C82, C83, C84, and N50 atoms were buried in the TMeQ[6] host, and the C81, C85, C86, C87, N49, and N51 atoms were located outside the TMeQ[6] host. The information obtained from the structure is in agreement with the ^1^H NMR analysis. As observed in the crystal structure, there is a N-H···O hydrogen bond between N52 of guest **2** and O21 of the TMeQ[6] host, and a N-H···O hydrogen bond between N53 and O16 of the portal O atoms of TMeQ[6], as well as a N-H···O hydrogen bond between N53 and O18 of the portal O atoms of TMeQ[6]. The nearby molecules create a 1D chain via the hydrogen bonds of the N atoms of guest **2** and the O atoms of water. Thus, guest **2** exhibited a straight chain, whereas guest **1** exhibited a back-folded geometry.

**Figure 6:**
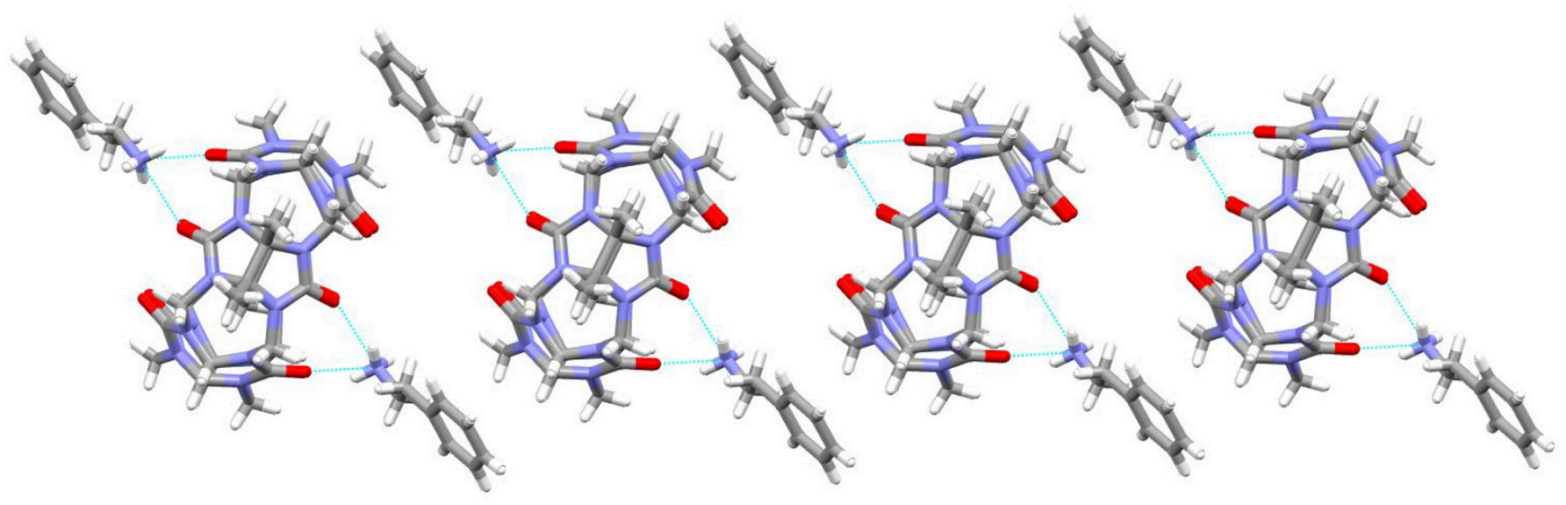
1D chain structure of TMeQ[6]@**3**. Solvent water molecules and tetrachloride cadmiumate anions have been excluded for lucidity. O, red; C, gray; N, light blue.

It was observed that the TMeQ[6]@**3** crystallized in a triclinic system with the P-1 space group. As shown in Figure 6, two guest **3** molecules exist on the portal of the TMeQ[6]. The N-H···O hydrogen bonds exist between the portal O atoms of TMeQ[6] and the N atoms of guest **3**. The structure suggests that guest **3** was found outside the TMeQ[6]; this discovery runs counter to the outcome of the evaluation of ^1^H NMR. The experimental outcomes indicate that the TMeQ[6]-guest inclusion states could be changed by altering the synthetic conditions. The cavity of TMeQ[6] is big enough to accommodate the guest **3** molecule in neutral aqueous solution, and guest **3** was found outside TMeQ[6], in an acidic environment and with [CdCl_4_]^2−^ anions. The hydrogen bond interaction and outer surface interaction of Q[*n*] has a pivotal part in producing the inclusion complex [20].

**Figure 7:**
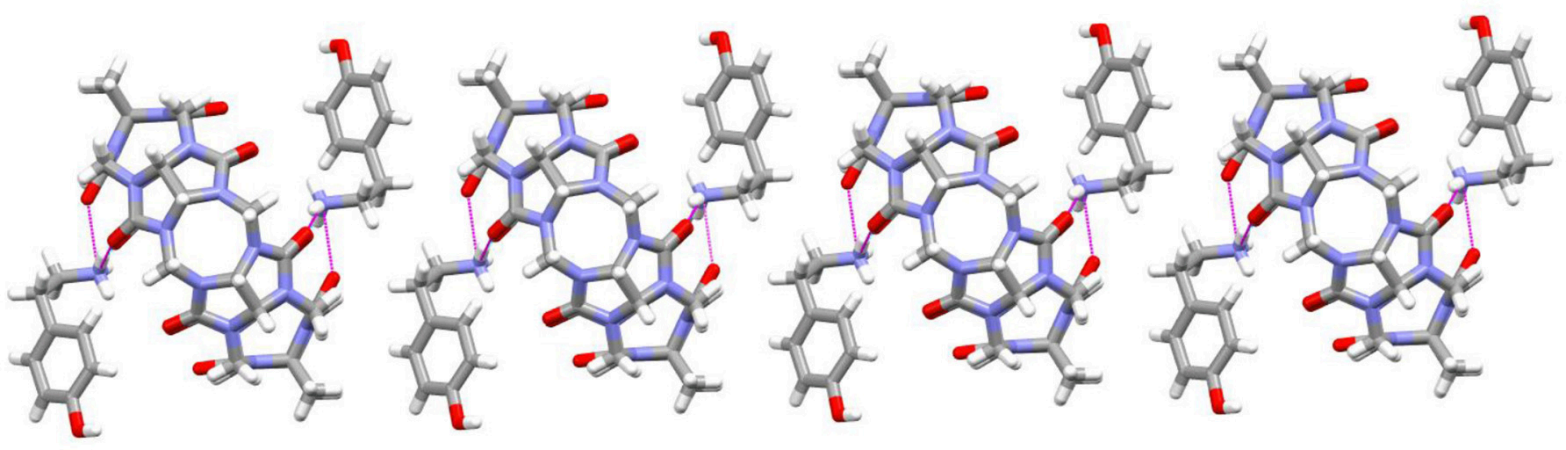
1D chain structure of TMeQ[6]@**4**. Solvent water molecules and tetrachloride cadmiumate anions have been excluded for lucidity. O, red; C, gray; N, light blue.

As revealed in Figure 7, the TMeQ[6]@**4** complex is made up of guest **4** found outside of the TMeQ[6] host. There are strong hydrogen bonds between the guest **4** N atoms and the O atoms of the TMeQ[6] host. Moreover, several fascinating hydrogen bonding interactions are present between guest **4** and the TMeQ[6] host, such as the C-H···O hydrogen bond between the TMeQ[6] methyl group and the portal carbonyl group of the other TMeQ[6], and the C-H···O hydrogen bond between the hydroxyl group of guest **4** and the portal carbonyl group of TMeQ[6], which leads to guest **4** being found outside of the TMeQ[6] host.

## Conclusion

The binding interactions of biogenic amines and TMeQ[6] in aqueous solution were examined by NMR spectroscopy, ITC, and MALDI-TOF mass spectrometry. The binding interactions of the biogenic amine guest and the TMeQ[6] host were examined in the solid state by X-ray crystallography, with the exception of guests **5** and **6**. As far as we know, the current evaluation is the initial one to structurally detail examples of cucurbit[*n*]uril and biogenic amine host–guest complexes. The results show that guests **1**, **2**, **3**, and **5** can be encapsulated into the TMeQ[6] host, whereas guests **4** and **6** are located outside of the TMeQ[6] host. The forces that facilitate the connection between the host TMeQ[6] and the guest biogenic amines include balanced hydrophobic, hydrogen bonding, and ion–dipole interactions. These outcomes will not just improve our understanding of the molecular identification of biogenic amines, but they may also be substantial for planning and generating novel macrocyclic complexes for biological recognition and replication.

## Experimental section

### Materials and methods

Spermine, spermidine, 2-phenethylamine, tyramine, histamine, and tryptamine were purchased. TMeQ[6] was prepared according to a literature method (Zhao et al., 2004). All NMR data were received in D_2_O at pD = 7 and pD = 3, respectively, the temperature is 298K, the concentration of the solution with guest is 1.00 × 10^−3^ mol/L, then TmeQ[6] was added into the solution gradually. MALDI-TOF mass spectra were taken on an ultrahigh-resolution Fourier transform ion cyclotron resonance (FT-ICR) mass spectrometer with α-cyano-4-hydroxycinnamic acid as matrix.

#### X-ray crystallography

All the crystals of complexes were grown from water. The crystal data was collected through the Bruker Apex II WITH CCD diffractometer. The crystal data was solved by the software SHELXS-204/7 (Sheldrick, 1997). CCDC numbers were 1815148, 1815149, 1816840, 1815151, respectively. These data can be obtained free of charge from The Cambridge Crystallographic Data Centre via www.ccdc.cam.ac.uk/data_request/cif.

#### Preparation of TMeQ[6]@1, TMeQ[6]@2, TMeQ[6]@3, and TMeQ[6]@4

To a solution of **1** (10.1 mg, 0.05 mmol) in HCl (1 M, 10 ml) was added TMeQ[6] (6.2 mg, 0.005 mmol), and CdCl_2_(5.50 mg, 0.03 mmol). The resulting reaction mixture was stirred for 5 min at 50°C and filtered. Slow solvent evaporation of the filtrate in air over a period of about 2 weeks provided white crystals of TMeQ[6]@**1** with yield of 1.8 mg (20%). Anal. Calcd for C_50_H_74_N_28_Cd_2_O_18_Cl_8_ (**1**): C, 32.22; H, 3.97; N, 21.05. Found: C, 32.19; H, 3.92; N, 21.00.

The two complexes were obtained following the method described above for TMeQ[6]@**1** except CdCl_2_ instead of ZnCl_2_. The yield based on TMeQ[6] for the three complexes are in the range 20-25%. Anal. Calcd for C_47_H_64.5_Zn_2_N_27_O_19.5_Cl_8_ (TMeQ[6]@**2**): C, 32.55; H, 3.72; N, 21.82. Found: C, 32.51; H, 3.69; N, 21.80. Anal. Calcd for C_48_H_56_CdN_25_O_13_Cl_4_ (TMeQ[6]@**3**): C, 39.89; H, 3.88; N, 24.24. Found: C, 39.95; H, 3.90; N, 24.30. Anal. Calcd for C_56_H_68_Cd_2_N_26_O_18_Cl_8_ (TMeQ[6]@**4**): C, 35.34; H, 3.58; N, 19.14. Found: C, 39.98; H, 3.62; N, 19.21.

## Supporting information

MALDI-TOF mass spectrum of inclusion complexes TMeQ[6]@**1**, TMeQ[6]@**2**, **T**MeQ[6]@**3**, TMeQ[6]@**4**, TMeQ[6]@**5**, and TMeQ[6]@**6**; ^1^H NMR spectra (400 MHz, pD = 3) of guest **1-6**; Electronic absorption and fluorescence emission spectra of guest **6**.

## Author contributions

LY, XX, and ZT write the manuscript. JK, FW, and QL analyse the data of the experiment. XW did the measurement. YZ synthesis the complex.

### Conflict of interest statement

The authors declare that the research was conducted in the absence of any commercial or financial relationships that could be construed as a potential conflict of interest.
