# Supplementary material for: Study on the Binding Interaction of the α,α′,δ,δ′-Tetramethylcucurbit[6]uril With Biogenic Amines in Solution and the Solid State
Source: Front Chem. 2018 Jul 17;6:289. doi: 10.3389/fchem.2018.00289 (PMC6057143; doi:10.3389/fchem.2018.00289)
Supplement: Supplementary file 1 [file Data_Sheet_1.doc]

**Study on the binding interaction of the cucurbit[n] uril with biogenic amines in solution and the solid state**

**Liguo Yang^a^, Jinglan Kan^b^, Xin Wang^a^, Yonghui Zhang^a^, Zhu Tao^c^, Qingyun Liu^d^, Fang Wang^a^, Xin Xiao^c^,***

**^a^** *College of Chemistry and Environmental Engineering, Anyang Institute of Technology, Anyang 455000, Henan, China*

**^b^** *College of Chemistry, Chemical Engineering and Materials Science, Collaborative Innovation Center of Functionalized Probes for Chemical Imaging in Universities of Shandong, Key Laboratory of Molecular and Nano Probes, Ministry of Education, Shandong Normal University, Jinan 250014, P. R. China*

**^c^** *Key Laboratory of Macrocyclic and Supramolecular Chemistry of Guizhou Province, Guizhou University, Guiyang 550025, China*

**^d^** *College of Chemistry and Environmental Engineering Shandong University of Science and Technology, Qingdao 266510, China*

**Table of contents**

1. ^1^H-^1^H COSY NMR spectrum (400 MHz) of inclusion complexes TMeQ[6]**@1,** TMeQ[6]**@2,** TMeQ[6]**@3** , TMeQ[6]**@4**, TMeQ[6]**@5** and TMeQ[6**]@6.**

2. ^1^H NMR spectra (400 MHz) of guest **3** in the absence (A) and presence of increasing equivalents of TMeQ[6](B-G) at 20°C.

3. MALDI-TOF mass spectrum of inclusion complexes TMeQ[6]**@1,** TMeQ[6]**@2,** TMeQ[6]**@3** , TMeQ[6]**@4**, TMeQ[6]**@5** and TMeQ[6**]@6.**

4. ^1^H NMR spectra (400 MHz, pD=3) of guest **1-6** in the absence (A) and presence of increasing equivalents of TMeQ[6](B-C) at 20°C.

5. Electronic absorption and fluorescence emission spectra of guest **6** in the absence (black) and presence (red) of TMeQ[6] with 1:1 stoichiometry.

6. ITC profile of TMeQ[6] with guests **1**, **2**, **3**, **5** and **6** at 298K.

7. Table S1. Binding constants *K_a_* (M^−1^) measured for the host-guest complexes of TMeQ[6] and compared to literature values for Q[6] and cucurbit[6]uril derivatives

8. Table S2. Selected hydrogen bonding (Å) for complexes **1**, **2**, **3** and **4.**

9. Refrence.


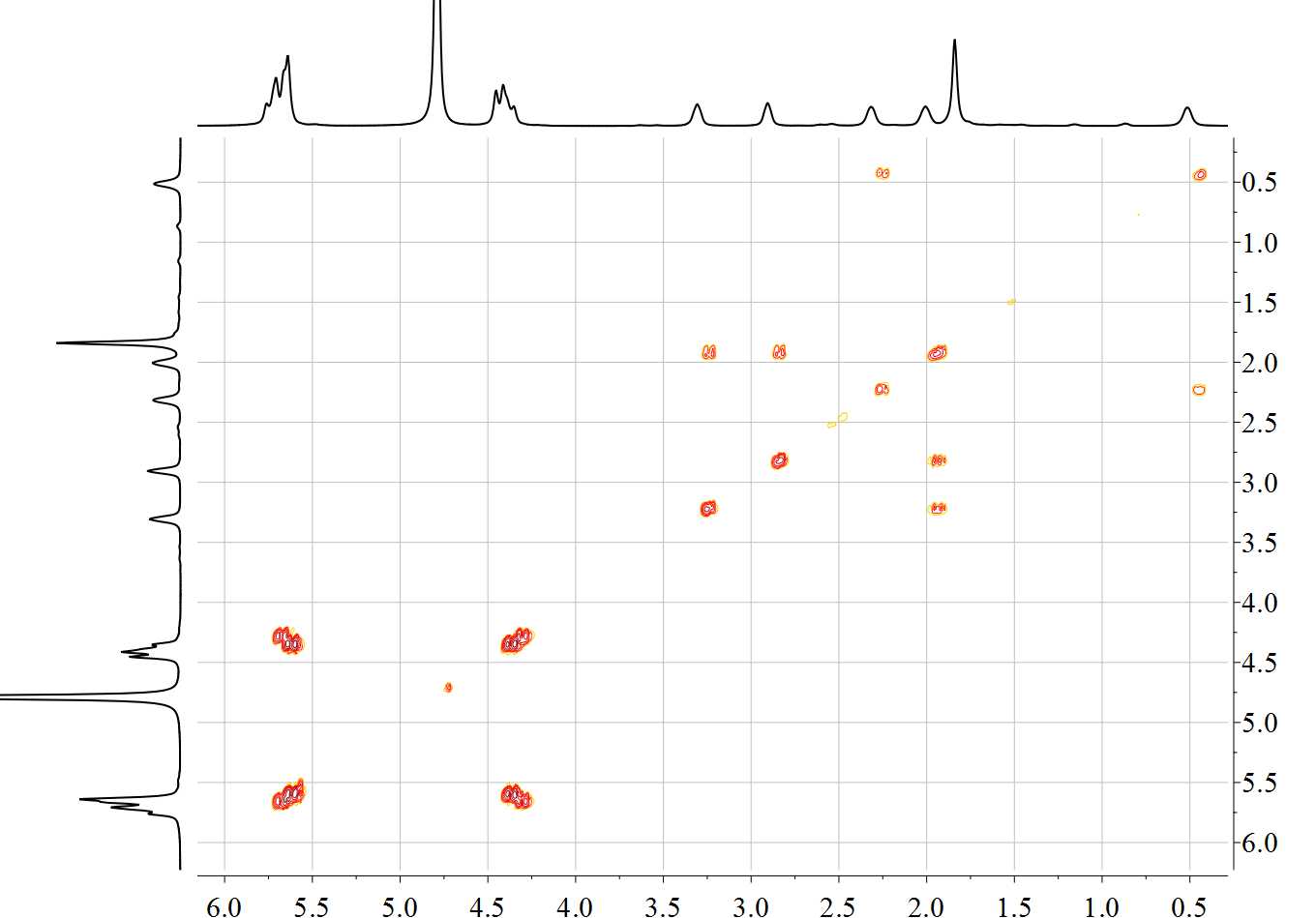


**Figure S1.** ^1^H-^1^H COSY NMR spectrum (400 MHz) of inclusion complexes TMeQ[6]**@1** recorded in D_2_O.


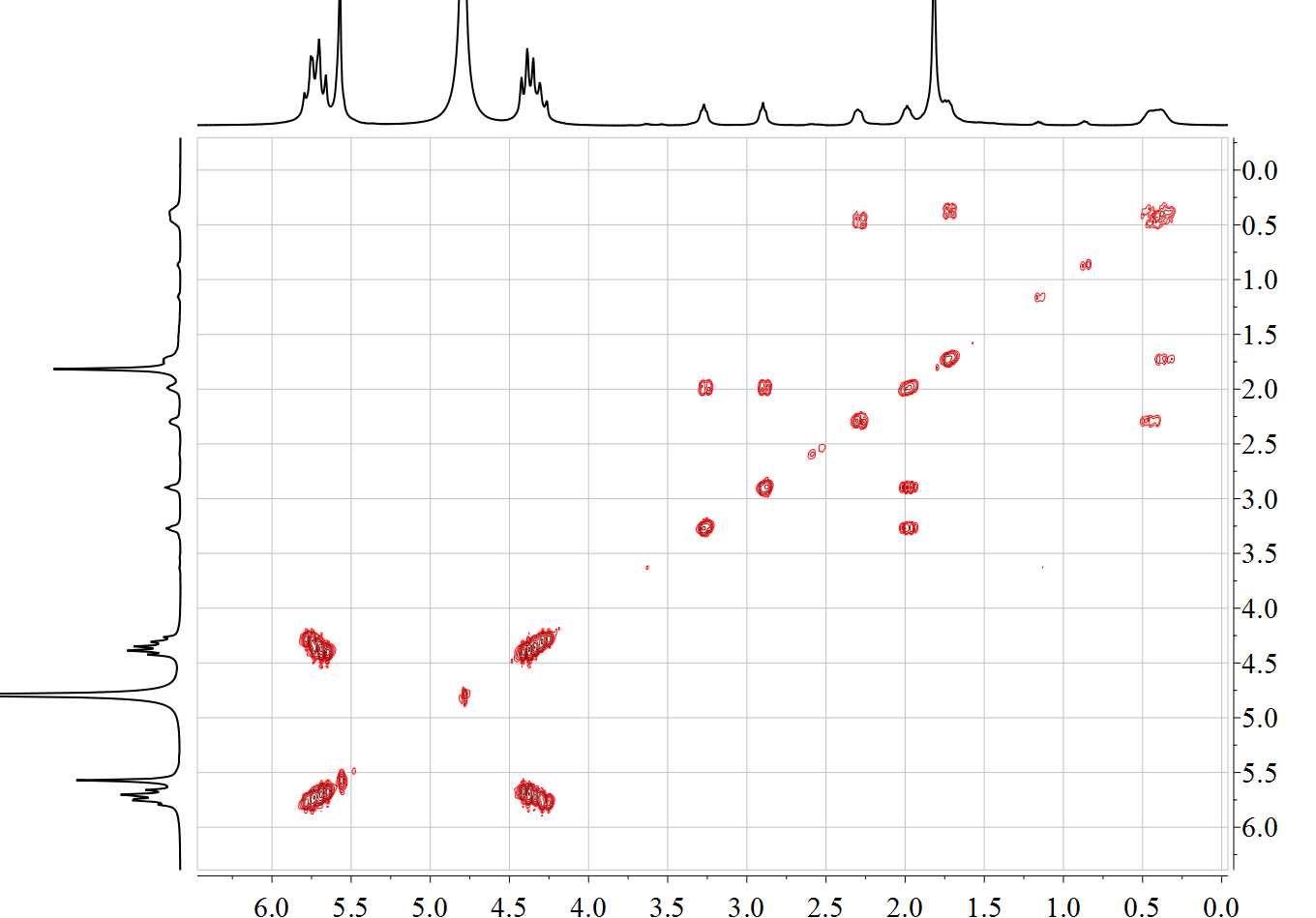


**Figure S2.** ^1^H-^1^H COSY NMR spectrum (400 MHz) of inclusion complexes TMeQ[6]**@2** recorded in D_2_O.


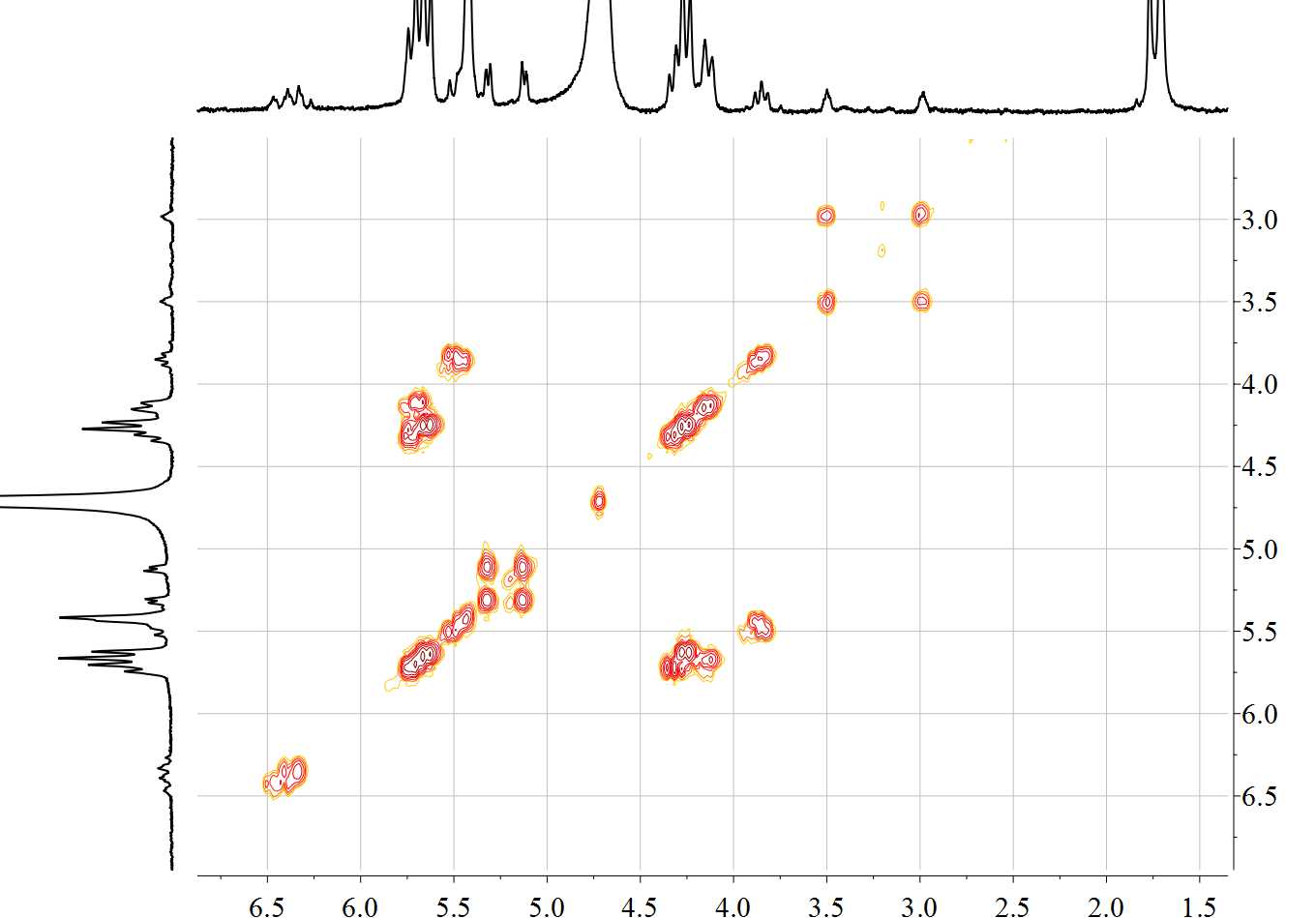


**Figure S3.** ^1^H-^1^H COSY NMR spectrum (400 MHz) of inclusion complexes TMeQ[6]**@3** recorded in D_2_O.


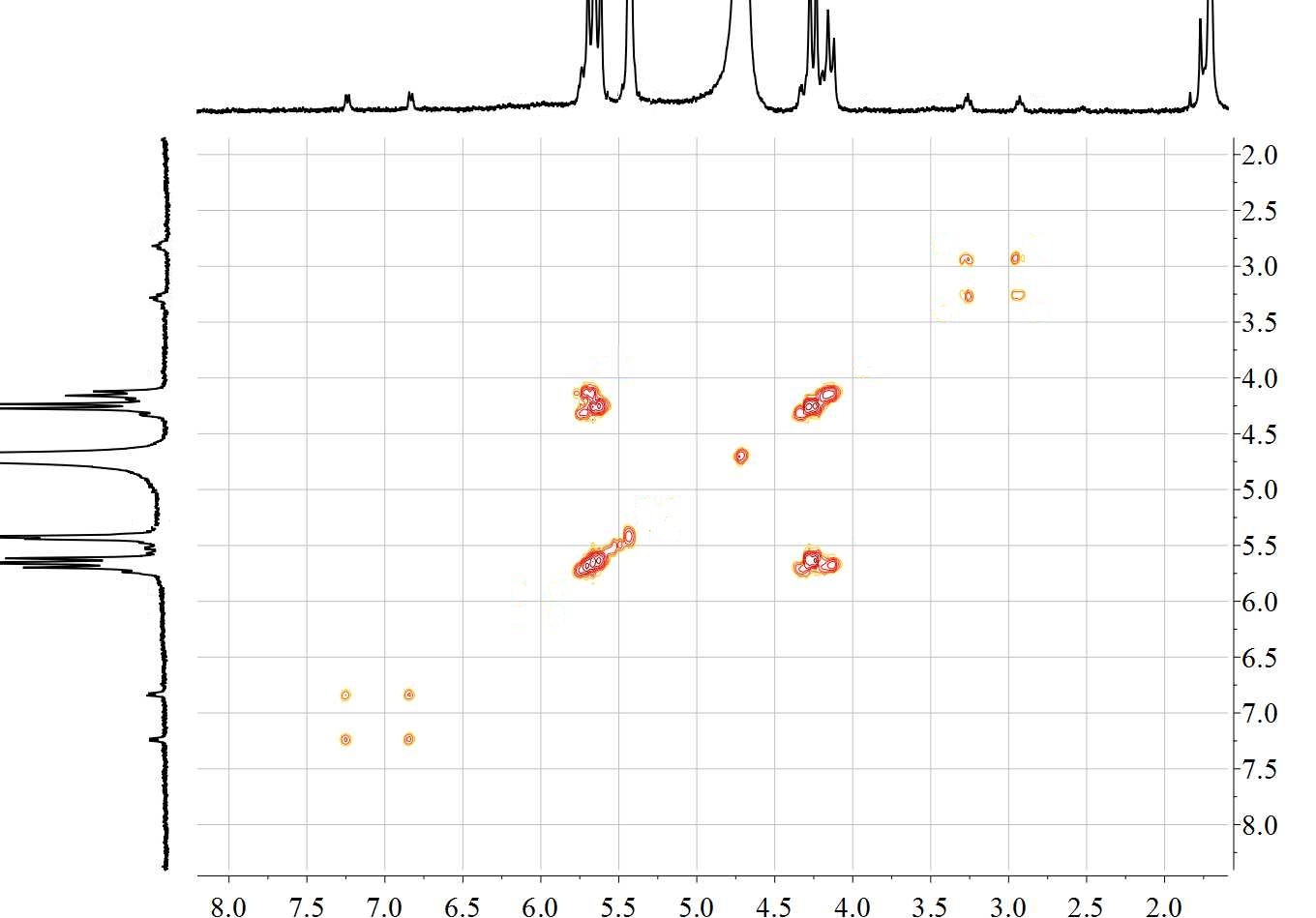


**Figure S4.** ^1^H-^1^H COSY NMR spectrum (400 MHz) of inclusion complexes TMeQ[6]**@4** recorded in D_2_O.


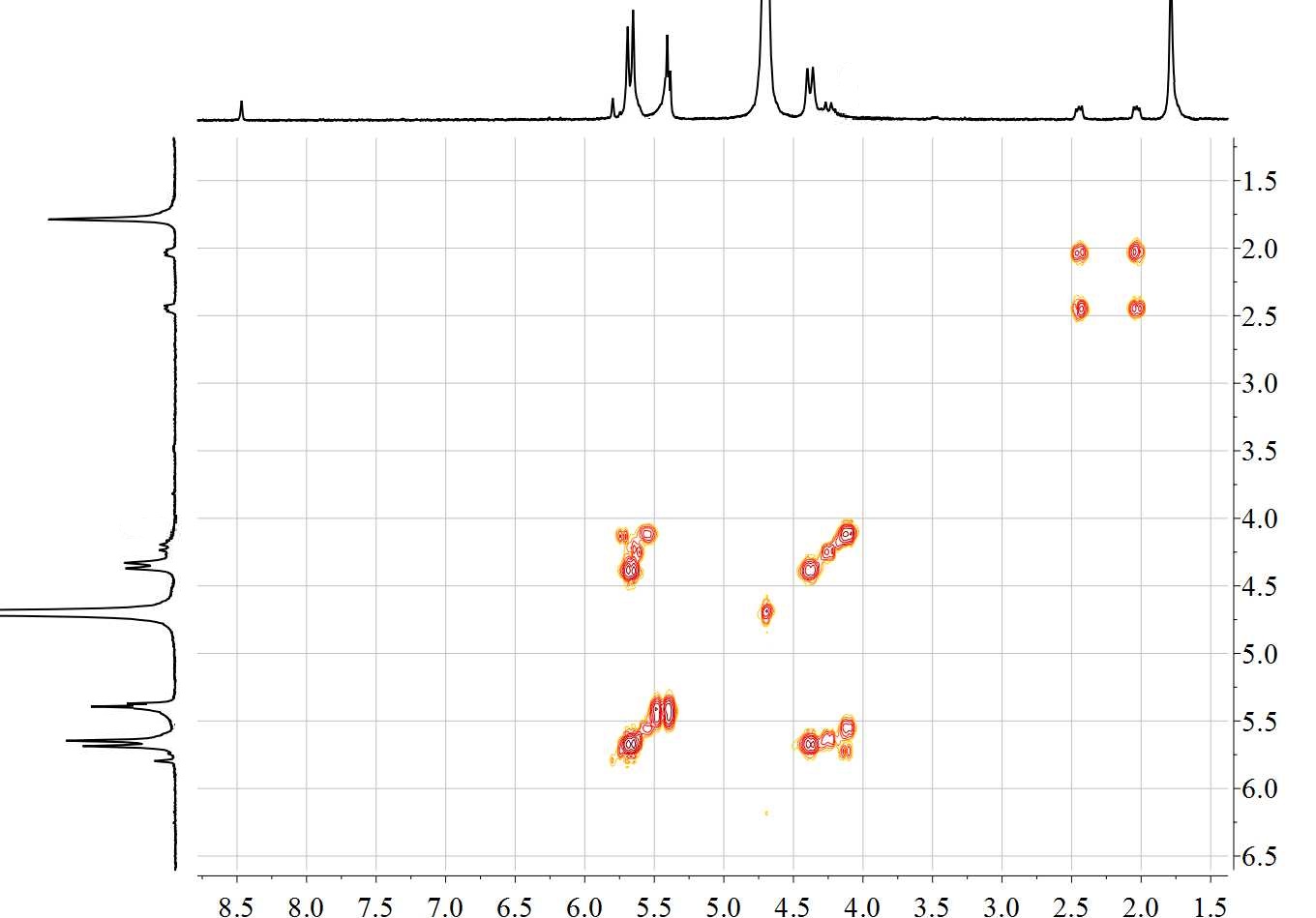


**Figure S5.** ^1^H-^1^H COSY NMR spectrum (400 MHz) of inclusion complexes TMeQ[6]**@5** recorded in D_2_O.


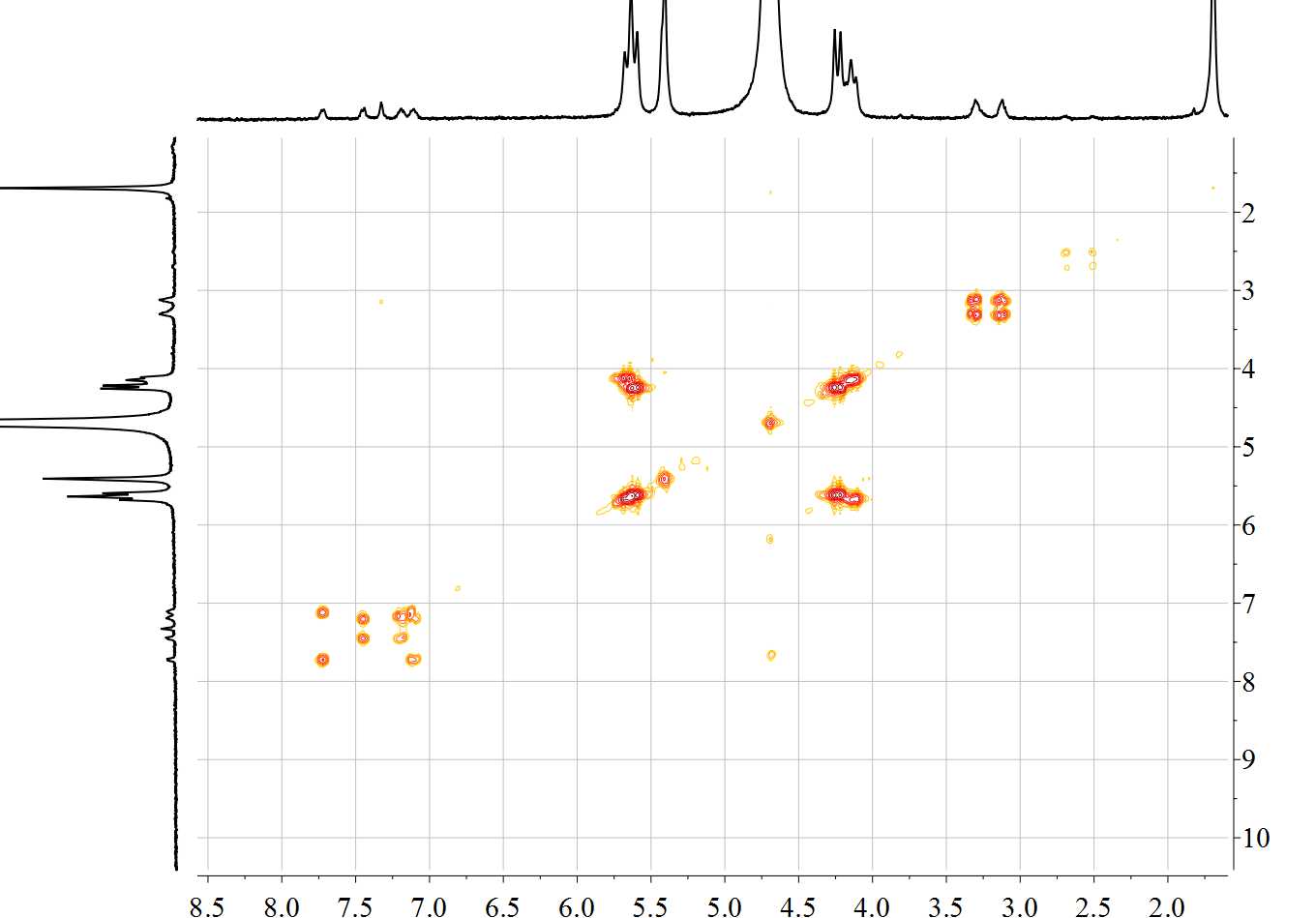


**Figure S6.** ^1^H-^1^H COSY NMR spectrum (400 MHz) of inclusion complexes TMeQ[6]**@6** recorded in D_2_O.


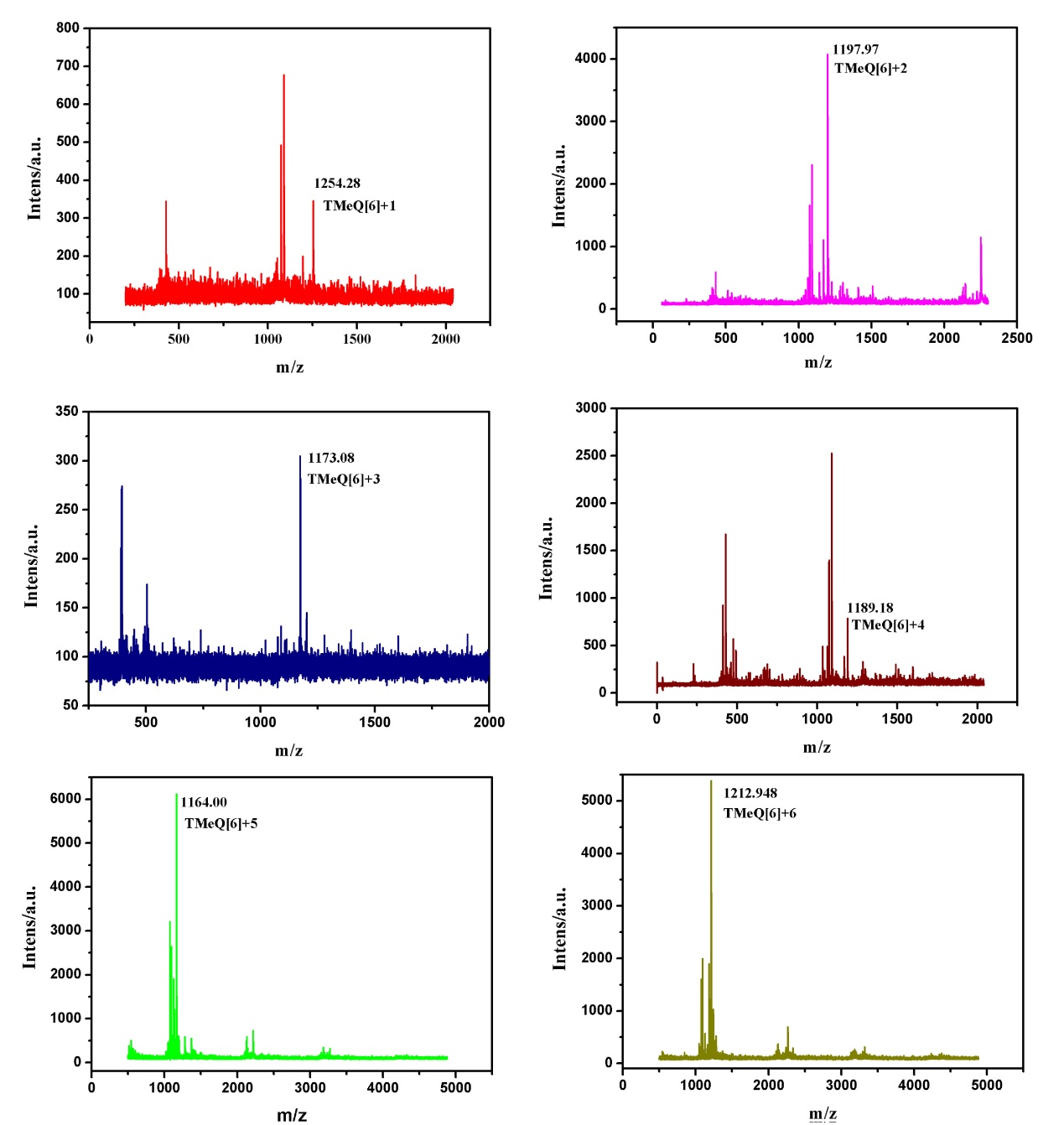


**Figure S7**. MALDI-TOF mass spectrum of inclusion complexes TMeQ[6]**@1,** TMeQ[6]**@2,** TMeQ[6]**@3** , TMeQ[6]**@4**, TMeQ[6]**@5** and TMeQ[6**]@6.**


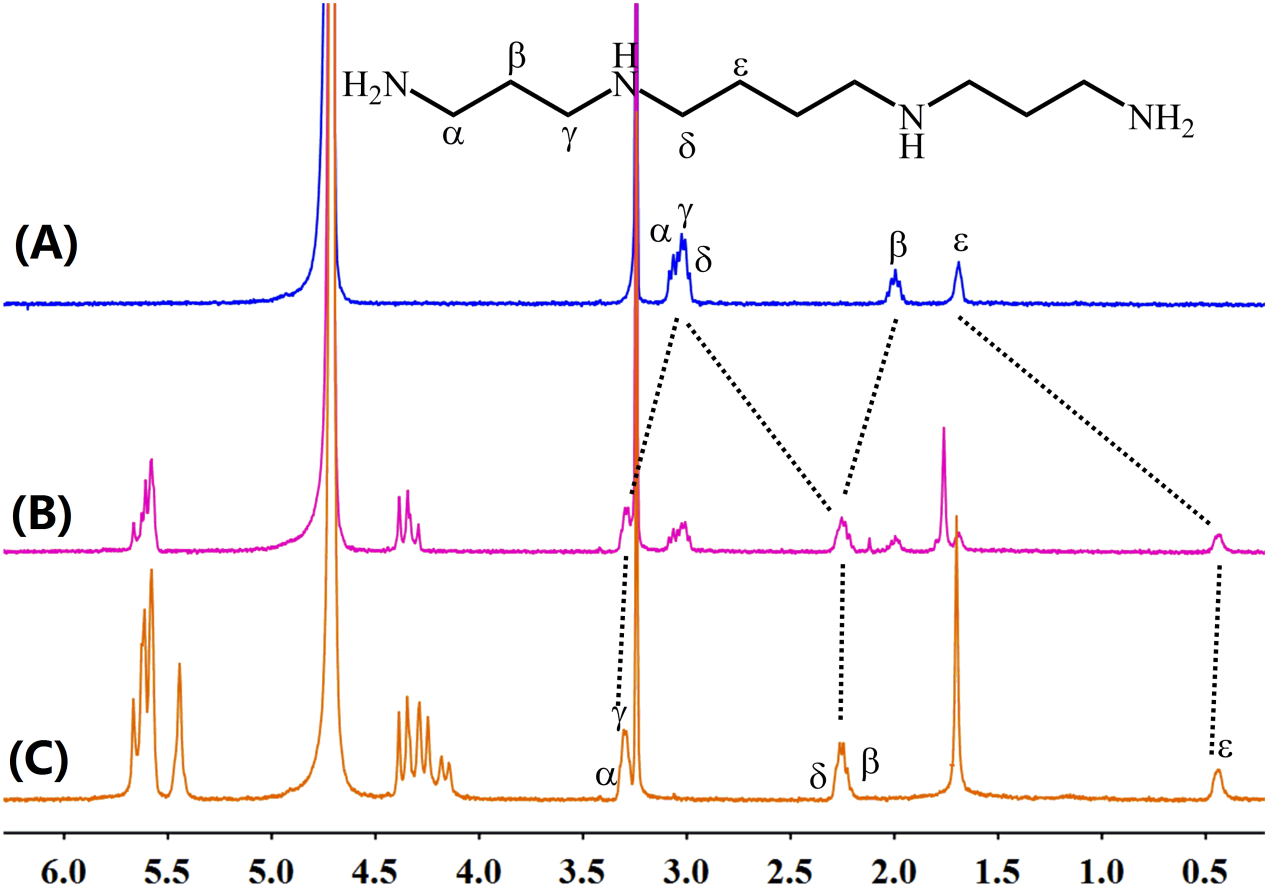


**Figure S8**. ^1^H NMR spectra (400 MHz, pD=3) of guest **1** in the absence (A) and presence of 0.78 (B), 1.1(C) equiv of TMeQ[6](B-C) at 20°C.


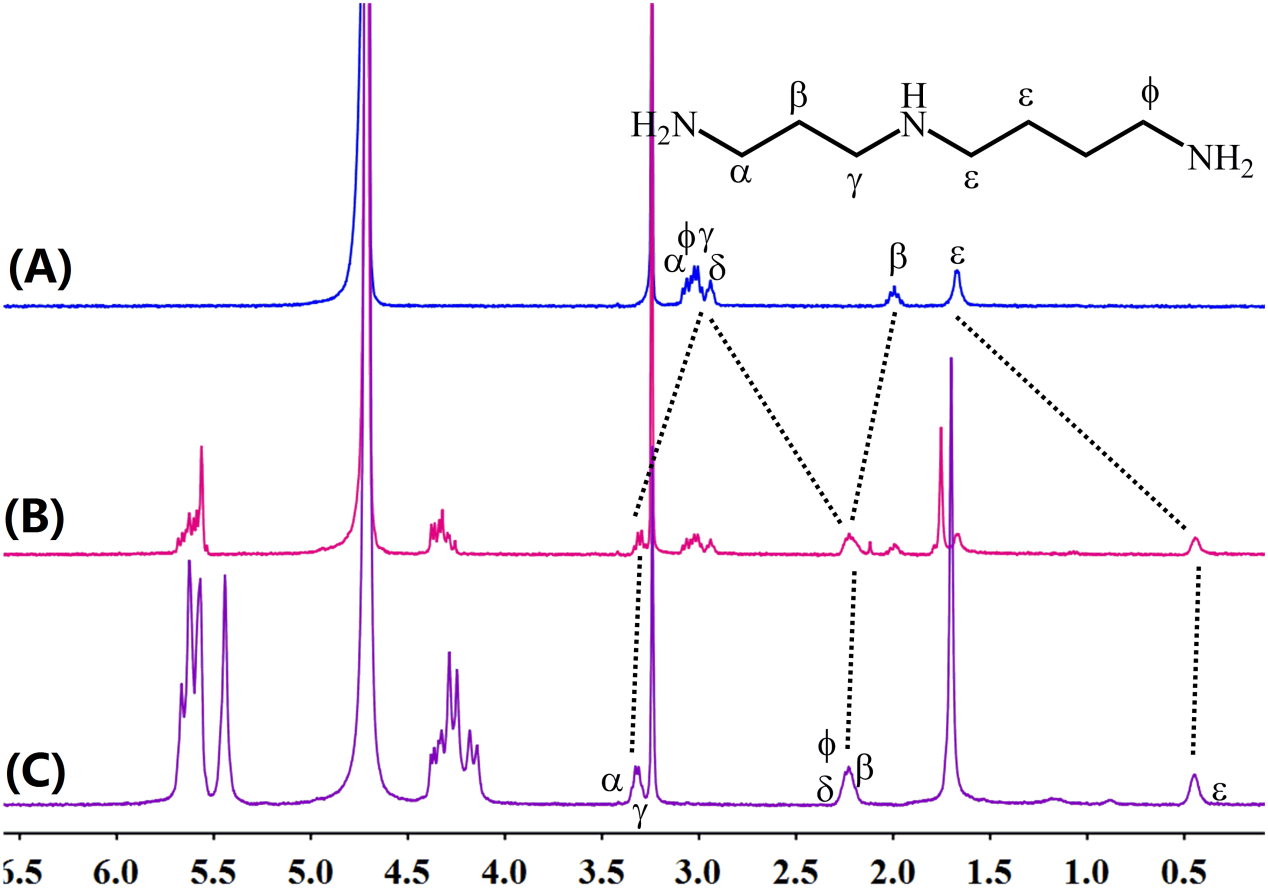


**Figure S9**. ^1^H NMR spectra (400 MHz, pD=3) of guest **2** in the absence (A) and presence of 0.82 (B), 1.05 (C) equiv of TMeQ[6](B-C) at 20°C.


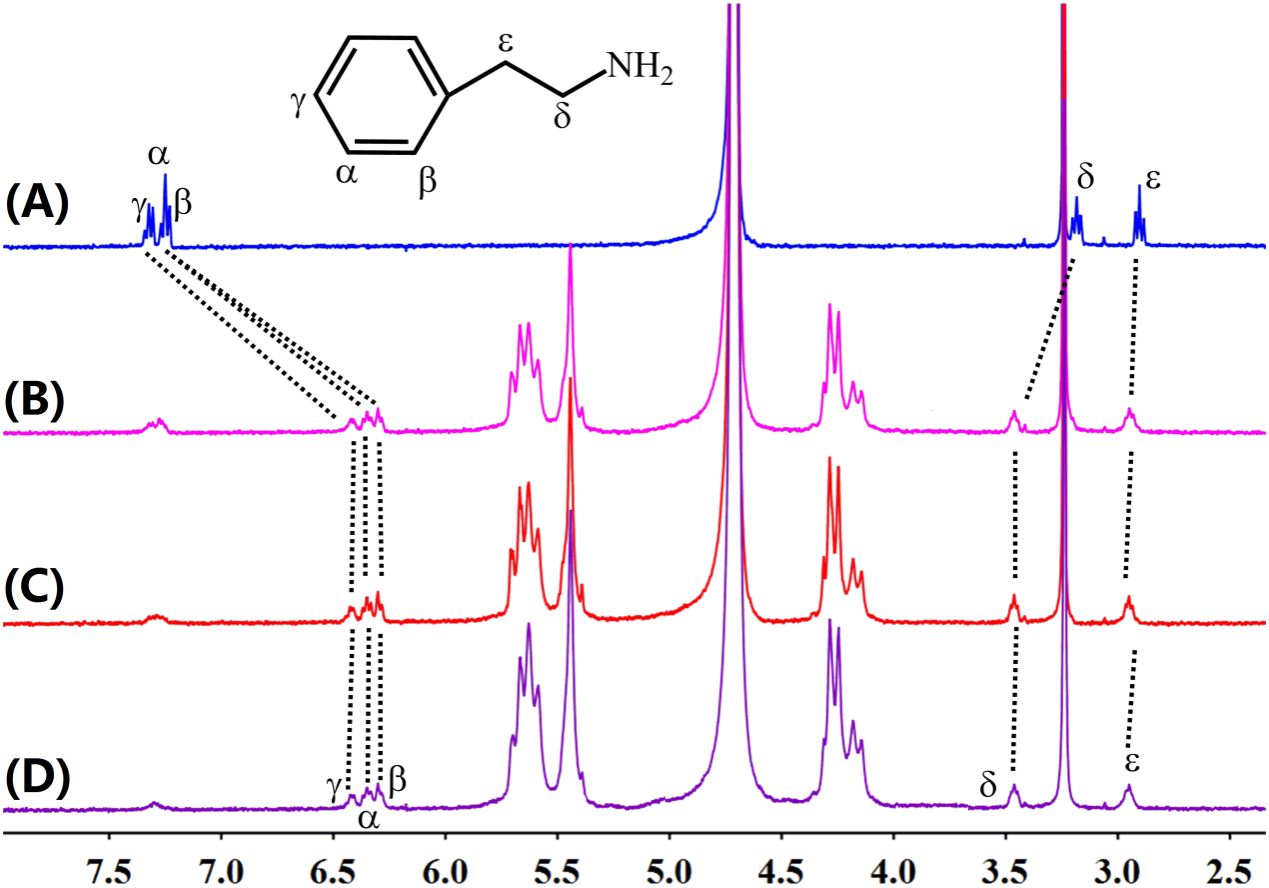


**Figure S10.** ^1^H NMR spectra (400 MHz, pD=3) of guest **3** in the absence (A) and presence of 0.57 (B), 0.75 (C), 1.2 (D) equiv of TMeQ[6](B-D) at 20°C.


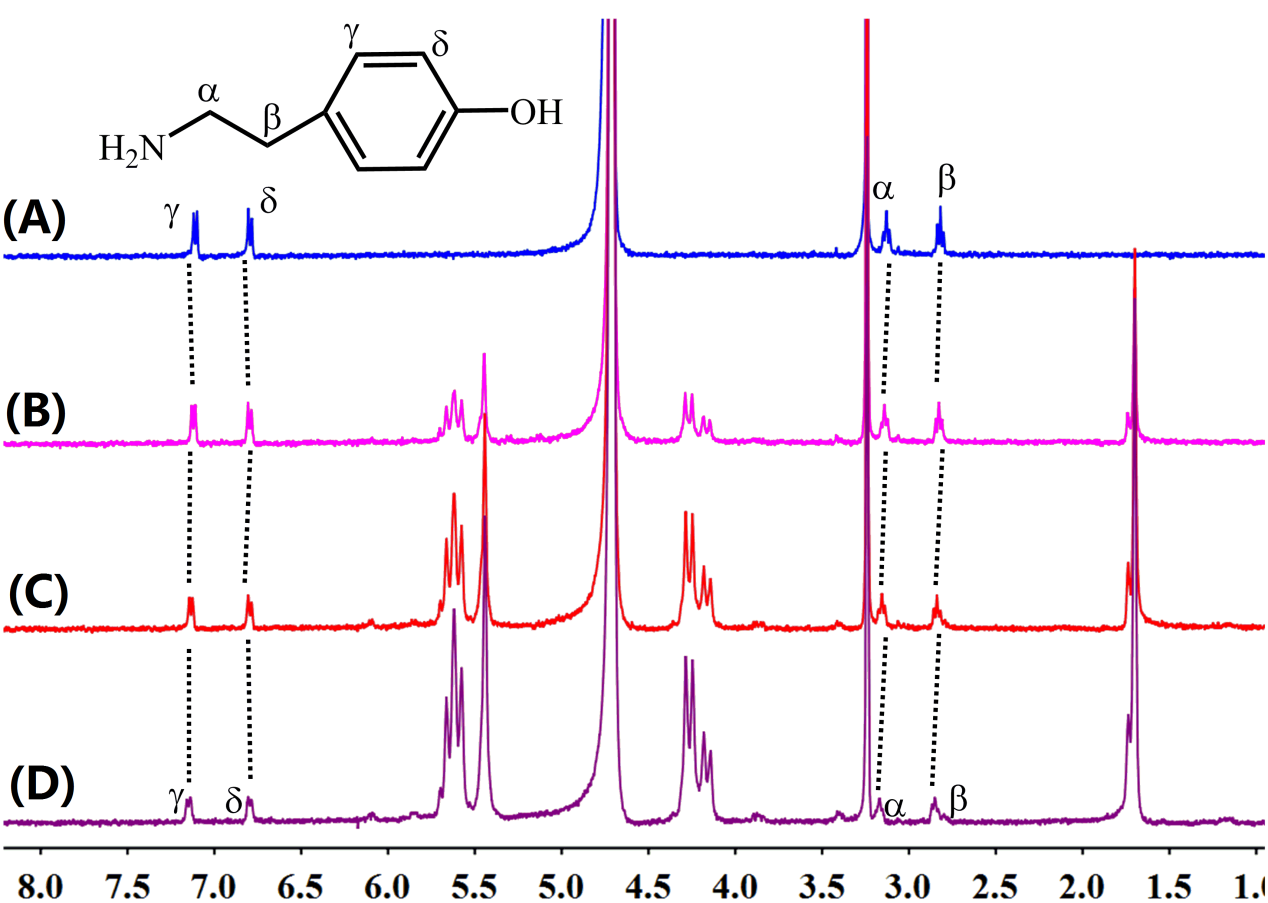


**Figure S11.** ^1^H NMR spectra (400 MHz, pD=3) of guest **4** in the absence (A) and presence of 0.46 (B), 0.85 (C), 1.2 (D) equiv of TMeQ[6](B-D) at 20°C.


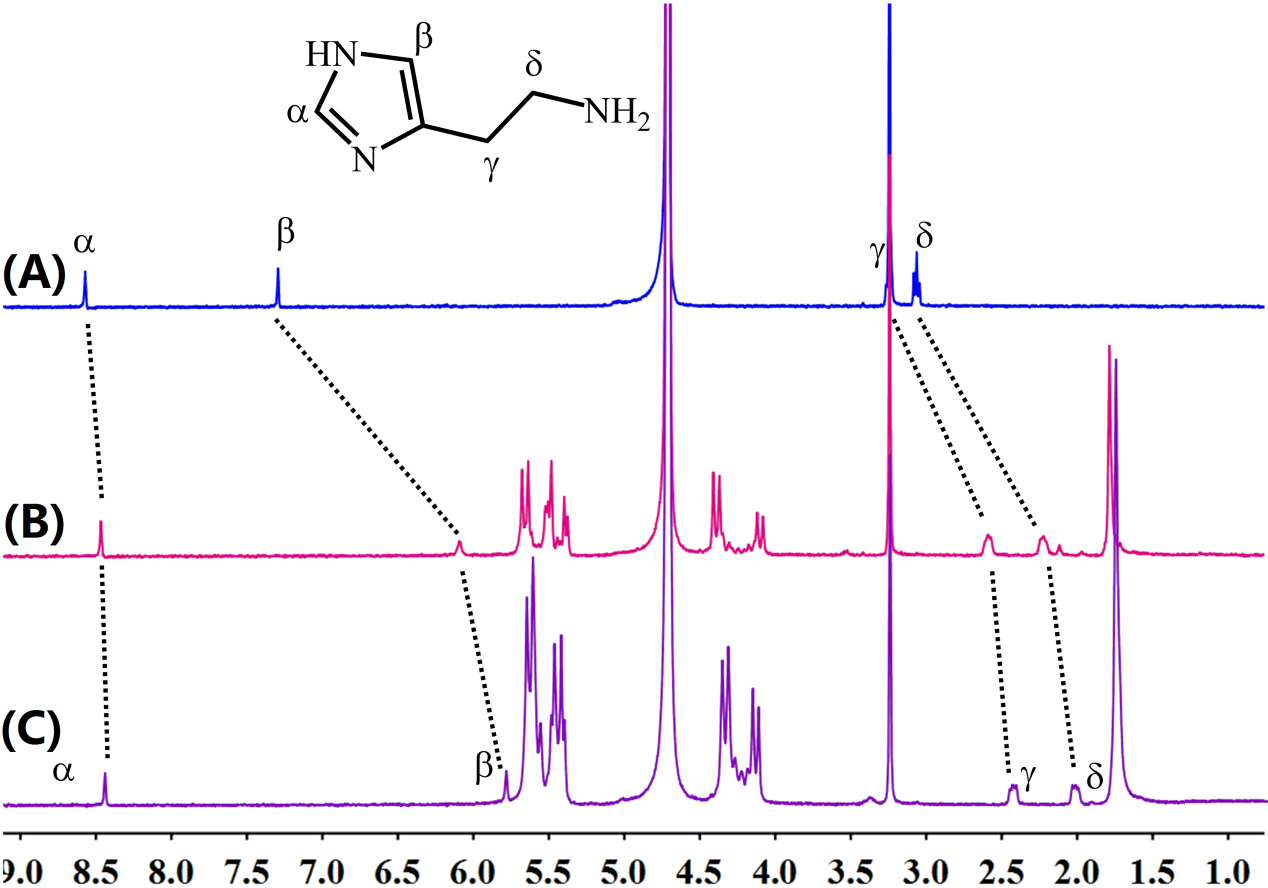


**Figure S12.** ^1^H NMR spectra (400 MHz, pD=3) of guest **5** in the absence (A) and presence of 0.78 (A), 1.1 (C) increasing equivalents of TMeQ[6](B-C) at 20°C.


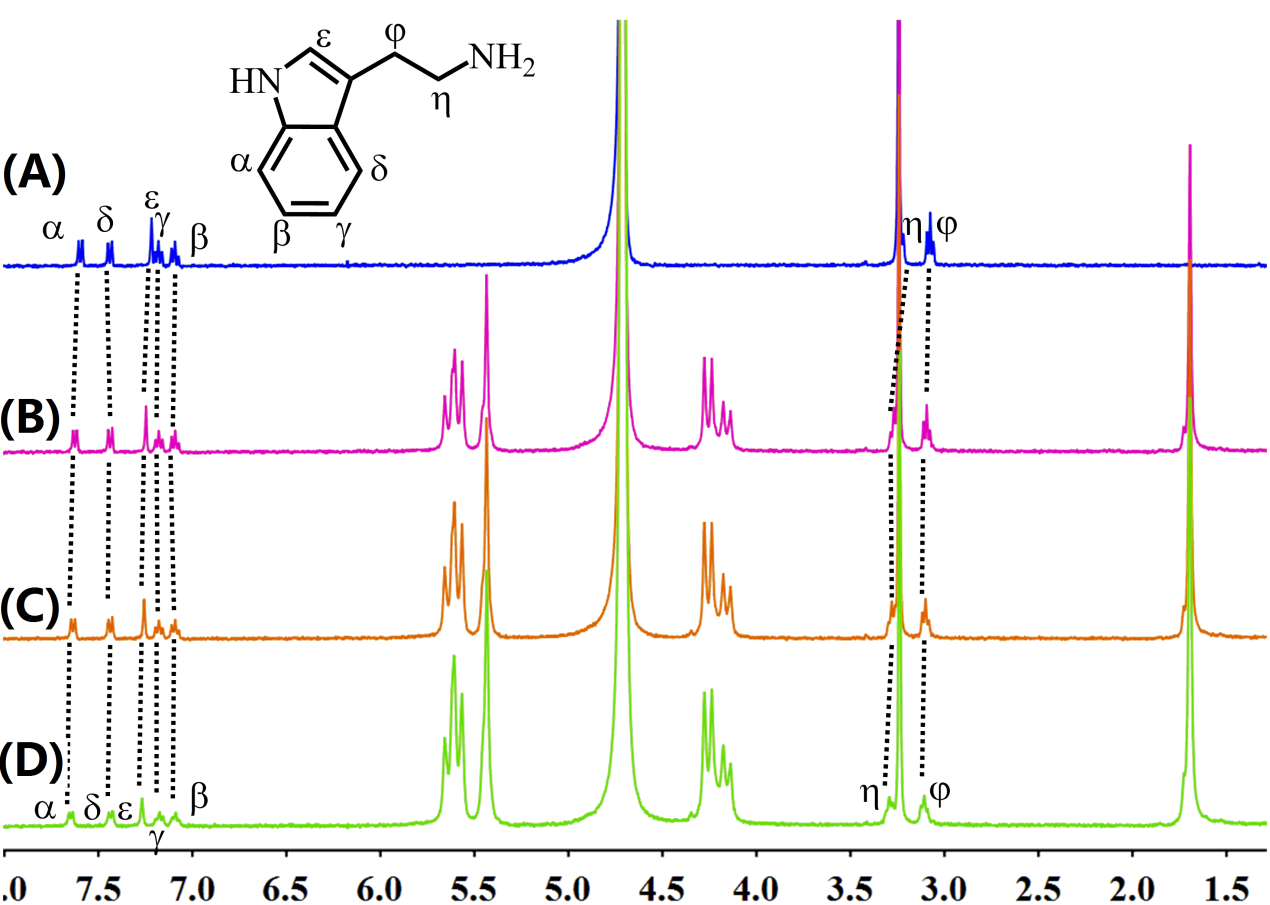


**Figure S13.** ^1^H NMR spectra (400 MHz, pD=3) of guest **6** in the absence (A) and presence of 0.56 (B), 0.88 (C), 1.1 (D) increasing equivalents of TMeQ[6](B-D) at 20°C.


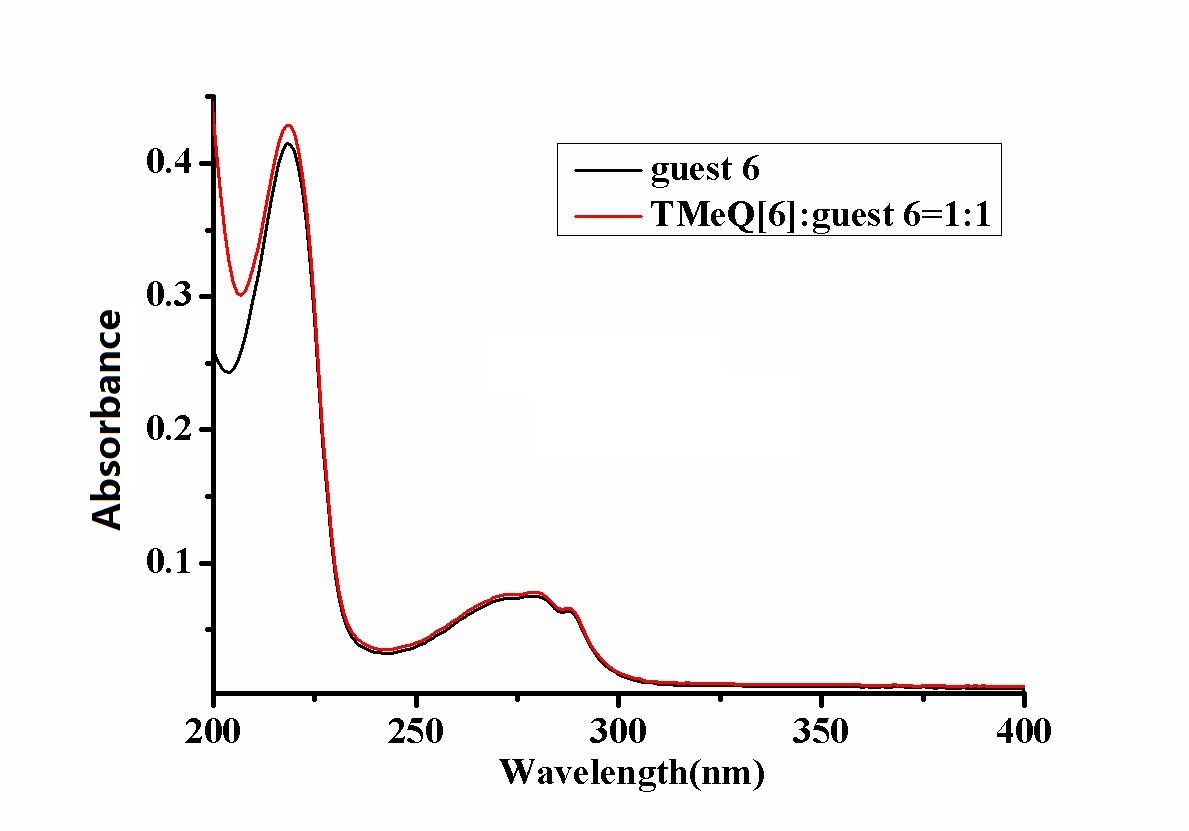


**Figure S14**. Electronic absorption spectra of guest **6**in the absence (black) and presence (red) of TMeQ[6] with 1:1 stoichiometry.


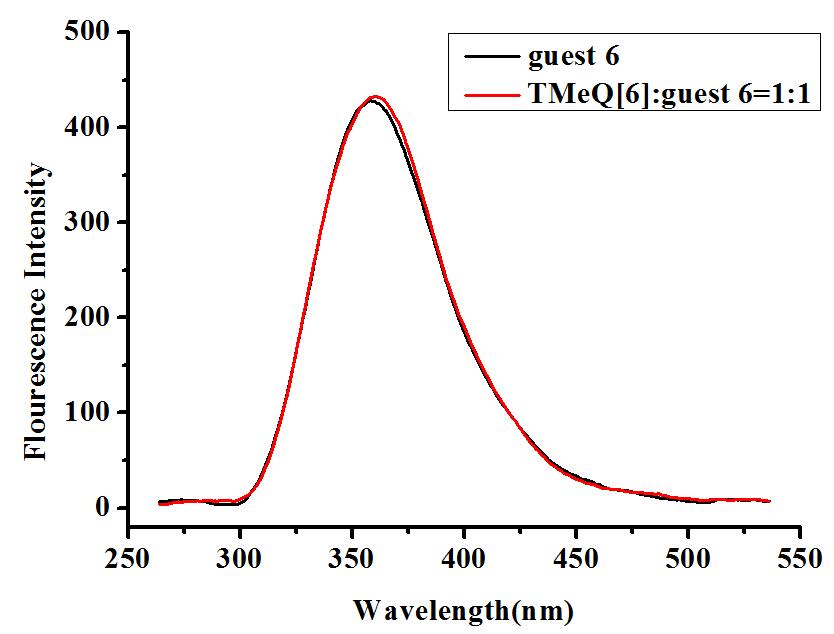


**Figure S15**. Fluorescence emission spectra of guest **6** in the absence (black) and presence (red) of TMeQ[6] with 1:1 stoichiometry.


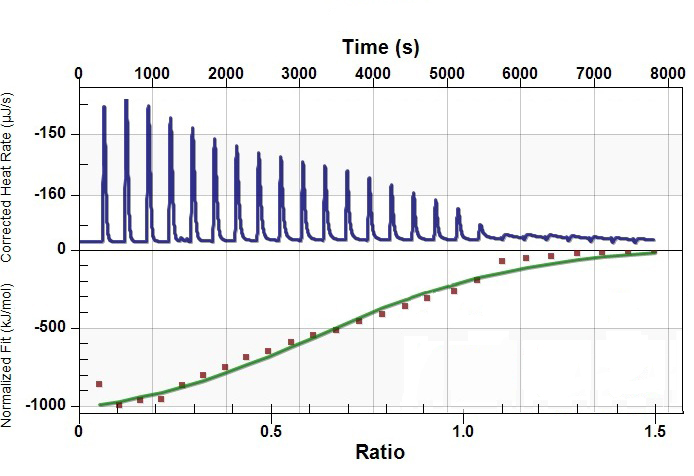


**Figure S16**. ITC profile of TMeQ[6] with guest **1** at 298 K.


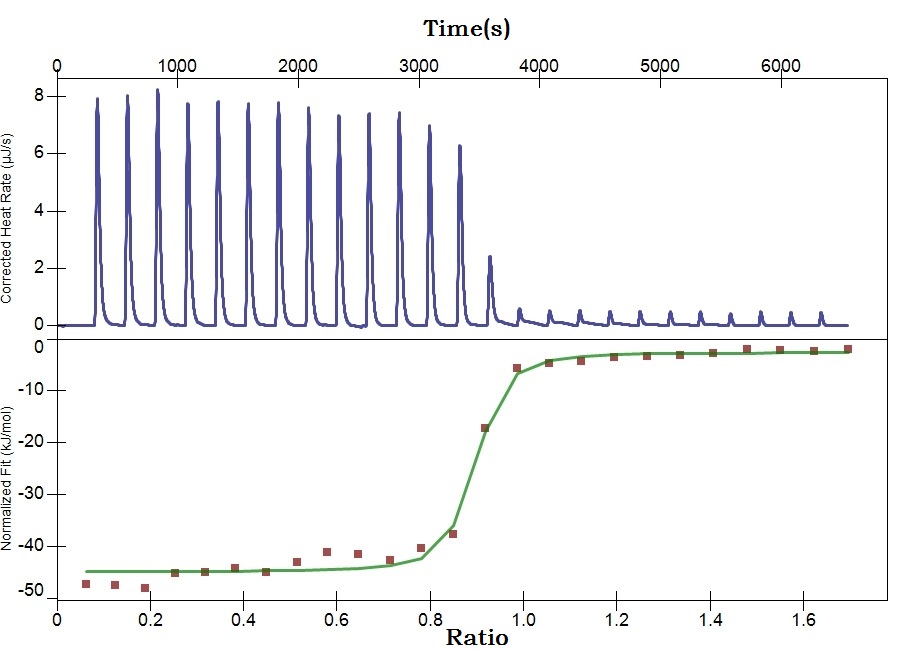


**Figure S17**. ITC profile of TMeQ[6] with guest **2** at 298 K.


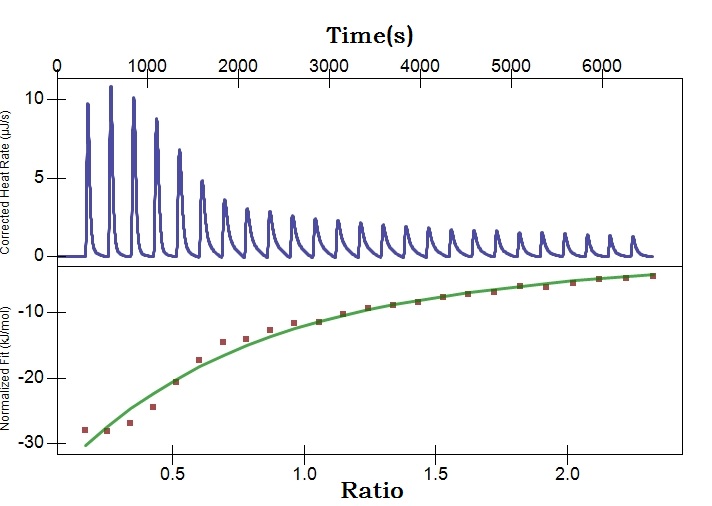


**Figure S18**. ITC profile of TMeQ[6] with guest **3** at 298K.


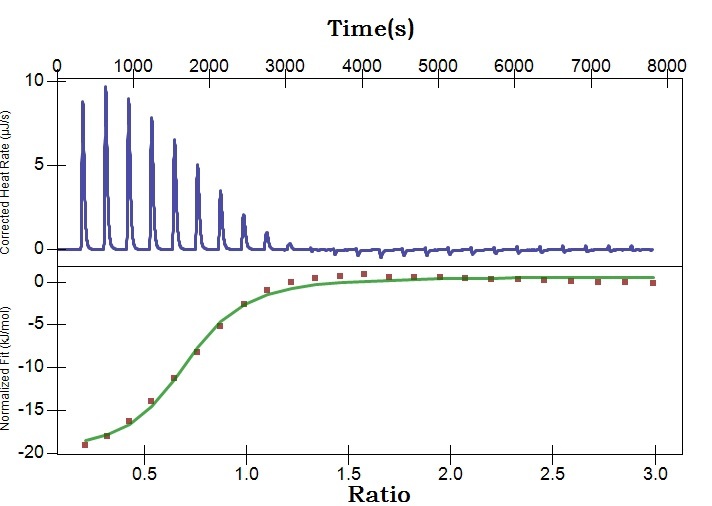


**Figure S19**. ITC profile of TMeQ[6] with guest **5** at 298 K.


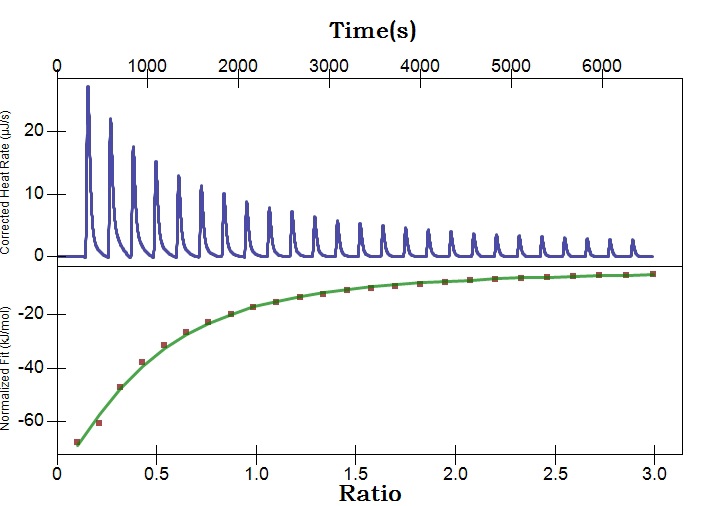


**Figure S20**. ITC profile of TMeQ[6] with guest **6** at 298K.

**Table S1** Binding constants *K_a_* (M ^−1^) measured for the host-guest complexes of TMeQ[6] and compared to literature values for Q[6] and cucurbit[6]uril derivatives

| Guest | TMeQ[6] | Q[6] ^[a]^ | CyH_6_Q[6] ^[b]^ | Other Q[6] derivative^[c]^ |
| --- | --- | --- | --- | --- |
| **1** | 4.45×10^7^ | 1.3×10^7^ | 3.4×10^12^ | 4.0×10^7^ |
| **2** | 9.97×10^6^ | \ | 4.8×10^11^ | \ |
| **3** | 5.5×10^3^ | \ | \ | \ |
| **5** | 1.59×10^5^ | \ | \ | 1.66×10^4^ |
| **6** | 1.23×10^4^ | \ | \ | \ |

^[a]^ Measured in1:1 HCO_2_H/H_2_O by Mock and co-workers(Lucas, et al., 2011).

^[b]^ Measured in pure water and added other competitive guest(Kim, et al., 2009).

^[c]^ Measured in 20 mM NaOAc buﬀered D_2_O at pD 4.74 by using ^1^H NMR analysis(Mock, et al., 1986).

**Table S2.** Selected hydrogen bonding (Å) for complexes **1**, **2**, **3** and **4.**

| **1** | | **2** | |
| --- | --- | --- | --- |
| N13Q-H13C···O6B | 2.256(3) | N54-H54B···O9 | 2.114(5) |
| N13Q-H13C···O5A | 2.263(3) | N50-H84A···O10 | 2.693(5) |
| N14P-H14A···O3 | 2.447(3) | N53-H91A···O16 | 2.573(5) |
| N14P-H14A···O4 | 2.053(3) | N53-H91B···O18 | 2.501(5) |
| **3** | | **4** | |
| N13O-H13A···O2 | 2.375(5) | N1-H1D···O9C | 2.124(3) |
| N13O-H13C···O5A | 2.372(5) | N1-H1D···O14F | 2.247(3) |
|  |  | N1-H1D···O11E | 2.201(3) |
|  |  |  |  |

**Refrence:**

Lucas, D., Minami, T., Iannuzzi, G., Cao, L. P., Wittenberg, J. B., Jr, A. P., Isaacs, L. (2011) Templated synthesis of glycoluril hexamer and monofunctionalized cucurbit [6] uril derivatives. *J. Am. Chem. Soc*. 133, 17966-17976. DOI: 10.1021/ja208229d

Kim, Y.; Kim, H.; Ko, Y. H.; Selvapalam, N.; Rekharsky, M. V.; Inoue, Y.; Kim, K.(2009) Soluble Cucurbit [6] uril Derivative in Pure Water: Isothermal Calorimetric, NMR, and X‐ray Crystallographic Study. Chem. Eur. J. 15, 6143-6151. DOI: 10.1002/chem.200900305

Mock, W. L., Shih, N. Y.(1986) Structure and selectivity in host-guest complexes of cucurbituril. J. Org. Chem. 51, 4440-4446. DOI: 10.1021/jo00373a018
